# Revision of the spider family Zodariidae (Arachnida, Araneae) in Iran and Turkmenistan, with seventeen new species

**DOI:** 10.3897/zookeys.1035.65767

**Published:** 2021-04-27

**Authors:** Alireza Zamani, Yuri M. Marusik

**Affiliations:** 1 Zoological Museum, Biodiversity Unit, University of Turku, FI-20014, Finland University of Turku Turku Finland; 2 Institute for Biological Problems of the North RAS, Portovaya Str.18, Magadan, Russia Institute for Biological Problems of the North Magadan Russia; 3 Department of Zoology & Entomology, University of the Free State, Bloemfontein 9300, South Africa University of the Free State Bloemfontein South Africa

**Keywords:** Ant-eating spiders, Aranei, Central Asia, Middle East, new record, taxonomy

## Abstract

Species of the spider family Zodariidae occurring in Iran and Turkmenistan are reviewed. Seventeen species of three subfamilies are described as new to science: *Lachesana kavirensis***sp. nov.** (♂, Qom; northern Iran), *L. perseus***sp. nov.** (♂, Alborz; northern Iran) (Lachesaninae), *Pax ellipita***sp. nov.** (♂♀, Kermanshah and Lorestan; western Iran), *P. leila***sp. nov.** (♂♀, Fars; southwestern Iran) (Storeninae), *Acanthinozodium armita***sp. nov.** (♂, Tehran; northern Iran), *A. atrisa***sp. nov.** (♂♀, Qazvin and Tehran; northern Iran), *A. diara***sp. nov.** (♂, Ilam and Lorestan; western Iran), *A. dorsa***sp. nov.** (♂♀, Fars; southern Iran), *A. elburzicum***sp. nov.** (♂♀, Tehran; northern Iran), *A. kiana***sp. nov.** (♂, Kurdistan; western Iran), *A. masa***sp. nov.** (♂, Kermanshah; western Iran), *A. niusha***sp. nov.** (♂♀, Fars, Isfahan and Markazi; central Iran), *A. ovtchinnikovi***sp. nov.** (♂, Mary; southeastern Turkmenistan), *A. parmida***sp. nov.** (♂, Isfahan; central Iran), *A. parysatis***sp. nov.** (♂♀, Ardabil and Qazvin; northern and northwestern Iran), *A. sorani***sp. nov.** (♂, East Azerbaijan and Kurdistan; northwestern and western Iran) and *Trygetus susianus***sp. nov.** (♀, Khuzestan; southwestern Iran) (Zodariinae). *Zodariellum* Andreeva & Tyshchenko, 1968, currently comprising only the type species (*Z. surprisum* Andreeva & Tyshchenko, 1968) is rediagnosed, with the following species being (re)transferred to it: *Z. asiaticum* (Tyshchenko, 1970) **comb. res.**, *Z. bactrianum* (Kroneberg, 1875) **comb. nov.**, *Z. bekuzini* (Nenilin, 1985) **comb. res.**, *Z. chaoyangense* (Zhu & Zhu, 1983) **comb. res.**, *Z. continentalis* (Andreeva & Tyshchenko, 1968) **comb. res.**, *Z. furcum* (Zhu, 1988) **comb. res.**, *Z. mongolicum* Marusik & Koponen, 2001 **comb. res.**, *Z. proszynskii* (Nenilin & Fet, 1985) **comb. res.**, *Z. nenilini* (Eskov, 1996) **comb. res.**, *Z. surprisum* Andreeva & Tyshchenko, 1968 **comb. res.**, *Z. schmidti* Marusik & Koponen, 2001 **comb. res.**, *Z. sytchevskajae* (Nenilin & Fet, 1985) **comb. res.** and *Z. volgouralensis* Ponomarev, 2007 **comb. res.** (all ex. *Zodarion*); out of these, *Z. proszynskii* Nenilin & Fet, 1985, previously known only from the type locality in Turkmenistan, is recorded from northeastern Iran for the first time. This paper raises the number of zodariids known from Iran to 22 species from seven genera (including the first Iranian records of *Acanthinozodium* Denis, 1966, *Pax* Levy, 1990 and *Zodariellum*) and those known from Turkmenistan to five species from five genera (including the first record of *Acanthinozodium* from this country). Regional distribution records of all species are mapped.

## Introduction

The spider family Zodariidae Thorell, 1881 comprises 1186 extant species in 87 genera and five subfamilies globally, as well as 11 species in nine genera known from fossils (Jocqué 1991; Dunlop et al. 2020; WSC 2021). Most of the species occur in the tropical and subtropical regions, with a few genera recorded from the Palaearctic (Jocqué and Dippenaar-Schoeman 2006). Despite their relatively high species richness, this family remains one of the most poorly documented groups of spiders in Iran and Turkmenistan. The first record of Zodariidae in Iran was provided by Ovtchinnikov et al. (2009), and currently, there are only six species belonging to four genera of this family known from this country (Zamani et al. 2021). Four species are known from Turkmenistan, three of which are endemics (Mikhailov 2013; WSC 2021). Recently, we had the opportunity to examine a relatively large collection of Iranian and Turkmen zodariids, in which 17 species are new to science, and two genera and one species in Iran and one genus in Turkmenistan are recorded for the first time. All of the species occurring in this region are surveyed, their distributions are mapped, and the new species are described and illustrated herein.

## Material and methods

Specimens were photographed using an Olympus Camedia E‐520 camera attached to an Olympus SZX16 stereomicroscope or to the eye piece of an Olympus BH2 transmission microscope, and a SEM JEOL JSM-5200 scanning electron microscope at the Zoological Museum of the University of Turku. Digital images were prepared using CombineZP image stacking software. Illustrations of internal genitalia were made after clearing them in a 10% KOH aqueous solution. Lengths of leg segments were measured on the dorsal side. Measurements of legs are listed as: total length (femur, patella, tibia, metatarsus, tarsus). All measurements are given in millimetres.

### Abbreviations

**AME** anterior median eye;

**ALE** anterior lateral eye;

**PME** posterior median eye;

**PLE** posterior lateral eye;

**RTA** retrolateral tibial apophysis.

### Depositories (with curators’ names in parentheses)

**MHNG**Muséum d’histoire naturelle, Genève, Switzerland (Peter J. Schwendinger);;

**MMUE**Manchester Museum of the University of Manchester, England (Dmitri V. Logunov);;

**NHMW**Naturhistorisches Museum Wien, Vienna, Austria (Christoph Hörweg);;

**NMP**Collection of the National Museum in Prague, Czech Republic (Petr Dolejš);;

**ZMMU**Zoological Museum of Moscow University, Moscow, Russia (Kirill G. Mikhailov).

## Taxonomy

### Family Zodariidae Thorell, 1881

#### Subfamily Lachesaninae

Taxon classificationAnimaliaAraneaeZodariidae

Jocqué, 1991

2B5E5090-6665-5E84-B16C-64DBCB5D7949

##### Comments.

The following four genera are currently considered in this subfamily, with the number of species known from each in parentheses: *Australutica* Jocqué, 1995 (6), *Antillorena* Jocqué, 1991 (4), *Lachesana* Strand, 1932 (8) and *Lutica* Marx, 1891 (4). *Lachesana* is the only one restricted to the western Palaearctic (Jocqué 2008; WSC 2021).

#### Genus Lachesana

Taxon classificationAnimaliaAraneaeZodariidae

Strand, 1932

E10481F9-F289-59A6-9088-F27E63C3A2FC

##### Type species.

*Lachesis perversa* Audouin, 1826 from Egypt.

##### Comments.

*Lachesana* spp. can easily be distinguished from other zodariids occurring in Iran and Turkmenistan by their large size (>10 mm *vs.* smaller) and numerous spines (>20 on each leg *vs.* lacking, or <15). So far, out of the eight species known from this genus, four are known solely on the basis of male specimens. Male palps are very similar to one another and differ mostly in the proportions of the tibial apophysis and the bulb.

#### Lachesana insensibilis

Taxon classificationAnimaliaAraneaeZodariidae

Jocqué, 1991

667578A9-87BD-5815-8C05-74BA9775A7AD

Fig. 32

Lachesana insensibilis Jocqué, 1991: 37, f. 59 (♂).

##### Comments.

This species is known from a single taxonomic entry, and unfortunately, the original description does not provide illustrations of the lateral view of the palp, making the identification of specimens from outside of the type locality (Saudi Arabia) highly questionable.

##### Records in Iran.

Khuzestan (Zamani et al. 2017) (Fig. 32).

##### Distribution.

Saudi Arabia, Israel, Iran, United Arab Emirates.

#### Lachesana kavirensis sp. nov.

Taxon classificationAnimaliaAraneaeZodariidae

75080B2F-5CE9-5599-9C92-74B037E542BE

http://zoobank.org/3BC489D8-1BD7-46C1-A4D2-BCB9A3BCDE58

**Figure 1.**
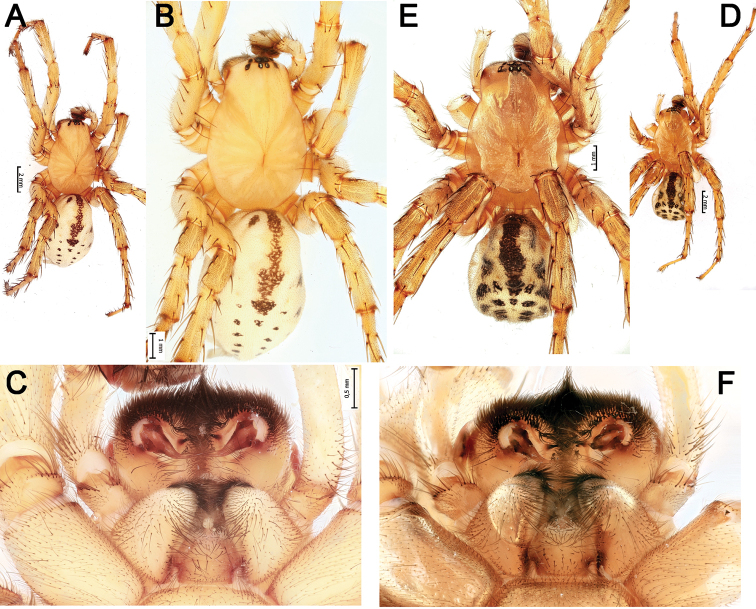
Habitus and chelicerae of male *Lachesana kavirensis* sp. nov. (**A–C**) and *L. perseus* sp. nov. (**D–F**) **A, B, D, E** habitus, dorsal **C, F** chelicerae, ventral.

Figs 1A–C , 3A–E , 4I , 32

##### Type material.

***Holotype*** ♂ (MHNG), Iran: *Qom Province*: Dasht-e Masileh, 34°47'N, 51°11'E, 11.2017 (M. Mirghazanfari).

##### Etymology.

The specific epithet is derived from ‘Kavir’, a Persian word for desert, referring to the occurrence of the species in the central deserts of Iran.

##### Diagnosis.

The male of *L. kavirensis* sp. nov. can be distinguished from the similar *L. dyachkovi* Fomichev & Marusik, 2019 by having a gently bent cheliceral fang (*vs.* bent over 90°, cf. Fig. 1C and fig. 15 in Fomichev and Marusik 2019). The shape of the RTA is also different from other species of the region (stalk with a gentle curve ventrally and tip straightly pointed; cf. Fig. 4I and Fig. 4F–H, J).

##### Description.

**Male.** Habitus as in Fig. 1A, B. Total length 11.55. Carapace 5.80 long, 2.60 wide at pars cephalica, 3.75 wide at pars thoracica. Eye sizes and interdistance of PMEs: AME: 0.23, ALE: 0.20, PME: 0.17, PLE: 0.23, PME–PME: 0.14. Carapace, sternum, labium, chelicerae and maxillae light brown. Chelicera (Fig. 1C) with dense, black setae and one promarginal tooth; fangs gently curved, almost straight. Legs the same color as carapace, without annulations and with numerous spines. Abdomen pale, dorsally with a longitudinal dark stripe and several small spots. Spinnerets slightly lighter than abdomen, uniform in color. Measurements of legs: I: 15.49 (4.47, 1.88, 3.33, 3.29, 2.52), II: 15.03 (4.20, 1.79, 3.02, 3.50, 2.52), III: 15.78 (4.08, 1.96, 2.48, 4.71, 2.55), IV: 18.30 (4.58, 2.07, 3.20, 5.24, 3.21).

Palp as in Figs 3A–E, 4I. RTA long, over 1.2 times longer than tibia, its widest part less than 2 times wider than stalk; cymbium with 2 spines in mesal proximal part; bulb elongate, 1.3 times longer than wide; base of embolus large, 1/3 of the tegulum's length; embolus filamentous, lying in chute formed by conductor.

**Female.** Unknown.

##### Distribution.

Known only from the type locality in Qom Province, northern Iran (Fig. 32).

#### Lachesana perseus sp. nov.

Taxon classificationAnimaliaAraneaeZodariidae

C18D05F5-6346-5EA4-8B4B-75AB8EAF40A2

http://zoobank.org/F3F99EF7-B5F4-42CB-AD3B-2404007AFBA2

**Figure 2.**
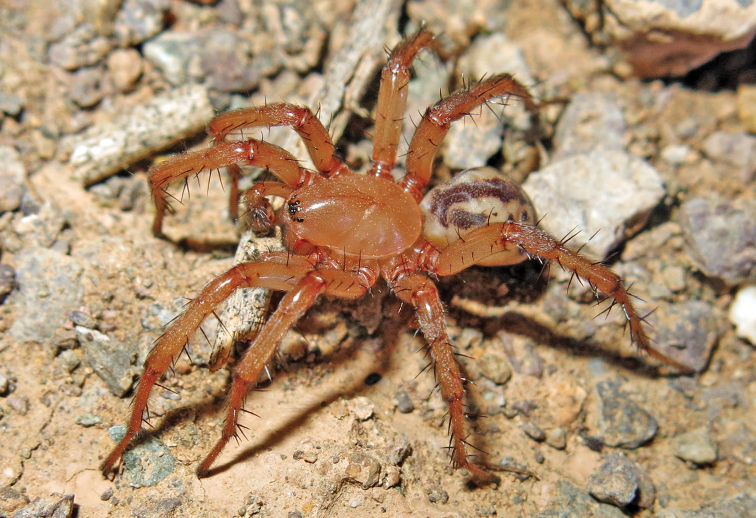
Live habitus of male *Lachesana perseus* sp. nov. Photo: A. Zamani.

**Figure 3.**
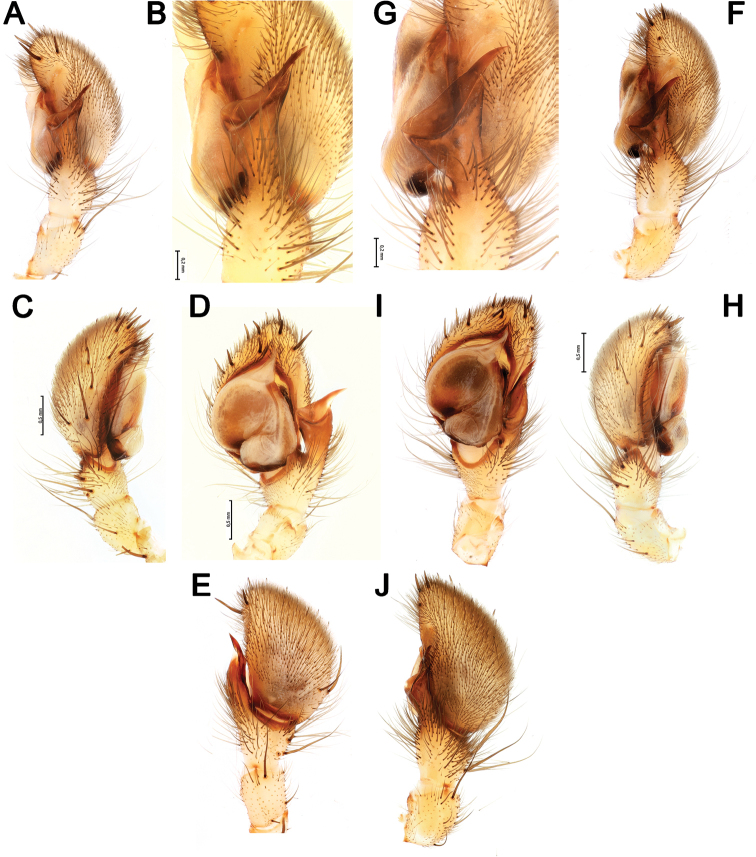
Male palps of *Lachesana kavirensis* sp. nov. (**A–E**) and *L. perseus* sp. nov. (**F–J**) **A, B, G, F** retrolateral **C, H** prolateral **D, I** ventral **E, J** dorsal.

**Figure 4.**
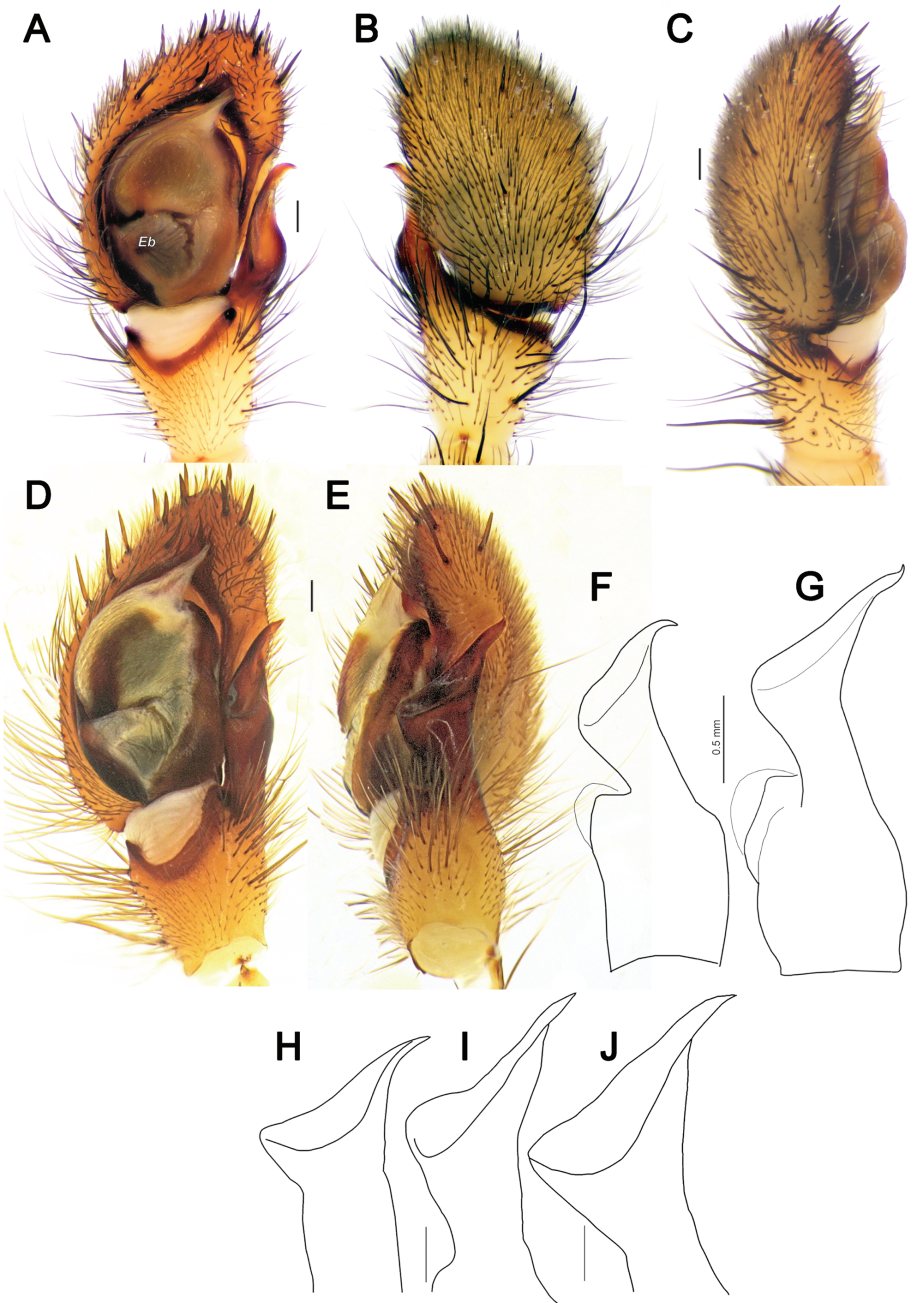
Male palps of *Lachesana dyachkovi* (**A–C, F**), *L. tarabaevi* (**D, E, G**), *L. blackwalli* (**H**), *L. kavirensis* sp. nov. (**I**) and *L. perseus* sp. nov. (**J**) **A, D** ventral **B, C, E** dorsal, prolateral and retrolateral **F–J** retrolateral tibial apophysis **A–G** reproduced after Fomichev and Marusik (2019)**H** illustrated after Özkütük et al. (2020). Scale bars: 0.2 mm, unless stated otherwise.

Figs 1D–F , 2 , 3F–J , 4J , 32

##### Type material.

***Holotype*** ♂ and ***paratype*** 1♂ (MHNG), Iran: *Alborz Province*: Eshtehard, Jaru, 35°44'N, 50°35'E, 10.2018 (A. Zamani).

##### Etymology.

The specific epithet refers to the legendary founder of Mycenae and of the Perseid dynasty in Greek mythology. Noun in apposition.

##### Diagnosis.

The new species differs from the similar *L. kavirensis* sp. nov. by the lack of spines in the proximal mesal part of the cymbium (*vs.* present; cf. Fig. 3C and 3H) as well as by the RTA with the terminal half almost as wide as the tibia (*vs.* 1.5 times thinner). The shape of the RTA readily distinguishes this species from congeners in the region (stalk abruptly bent basally and tip gently curved; cf. Fig. 4J and 4F–I).

##### Description.

**Male** (holotype). Habitus as in Figs 1D, E, 2. Total length 10.50. Carapace 5.60 long, 2.60 wide at pars cephalica, 3.88 wide at pars thoracica. Eye sizes and interdistance of PMEs: AME: 0.19, ALE: 0.19, PME: 0.16, PLE: 0.19, PME–PME: 0.18. Carapace, sternum, labium, chelicerae and maxillae light brown. Chelicera (Fig. 1F) with dense, black setae and one promarginal tooth; fangs curved. Legs the same color as carapace, without annulations and with numerous spines. Abdomen pale, dorsally with a longitudinal dark stripe and several large spots. Spinnerets grayish, uniform in color. Measurements of legs: I: 16.42 (4.69, 2.02, 3.61, 3.56, 2.54), II: 15.48 (4.22, 2.01, 3.19, 3.53, 2.53), III: 16.25 (4.46, 1.96, 2.51, 4.66, 2.66), IV: 19.39 (4.87, 2.32, 3.29, 5.66, 3.25).

Palp as in Figs 3F–J, 4J. Tibial apophysis more than 1.2 times longer than tibia, widest part over 2 times wider than stalk; cymbium lacking spines in proximal mesal part; bulb elongate, 1.3 times longer than wide; base of embolus large, 1/3 of the tegulum's length; embolus filamentous, lying in chute formed by conductor.

**Female.** Unknown.

##### Distribution.

Known only from the type locality in Alborz Province, northern Iran (Fig. 32).

### Subfamily Storeninae Simon, 1893

#### Genus Pax

Taxon classificationAnimaliaAraneaeZodariidae

Levy, 1990

5E0B26BF-C08A-592C-A10A-3AA52539B4A6

##### Type species.

*Habronestes libani* Simon, 1873 from Lebanon.

##### Comments.

This is a small genus with seven species distributed exclusively in the Middle East from Turkey and Israel to Iran (including the two new species described below, representing the first record of this genus in this country). Members of this genus can be easily distinguished from all other zodariids found in the study area by the ovoid carapace lacking a distinct separation between the cephalic and thoracic parts and also by males having a modified cymbium.

#### Pax ellipita sp. nov.

Taxon classificationAnimaliaAraneaeZodariidae

3D04C913-4C33-57AF-827C-326761DF8161

http://zoobank.org/D52287DC-6ED8-4E56-B435-F5CB56650469

**Figure 5.**
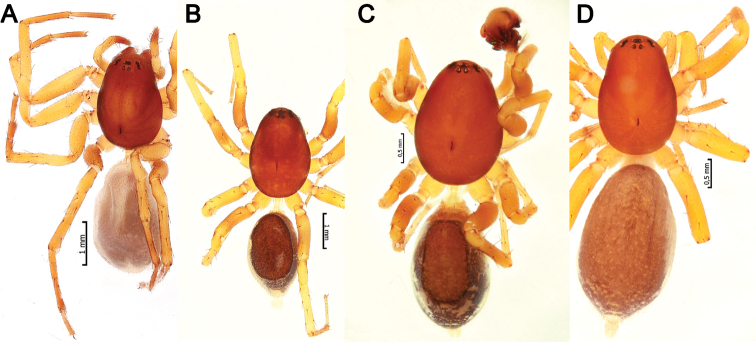
Dorsal habitus of *Pax ellipita* sp. nov. (**A, B**) and *P. leila* sp. nov. (**C, D**) **A, D** females **B, C** males.

**Figure 6.**
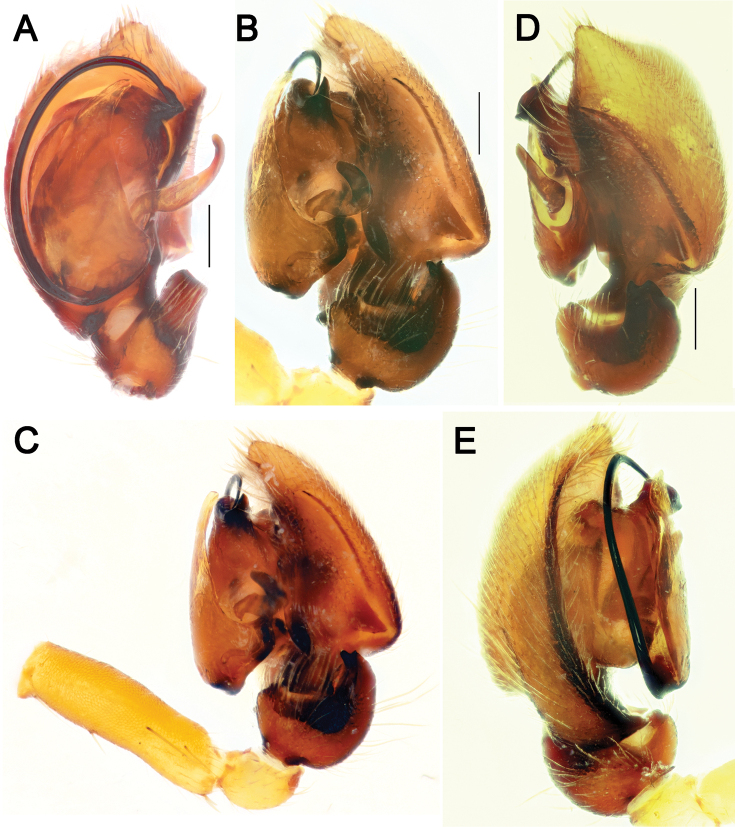
Male palp of *Pax ellipita* sp. nov. **A** ventral **B, C** retrolateral **D, E** retrodorsal and prolateral. Scale bars: 0.2 mm.

**Figure 7.**
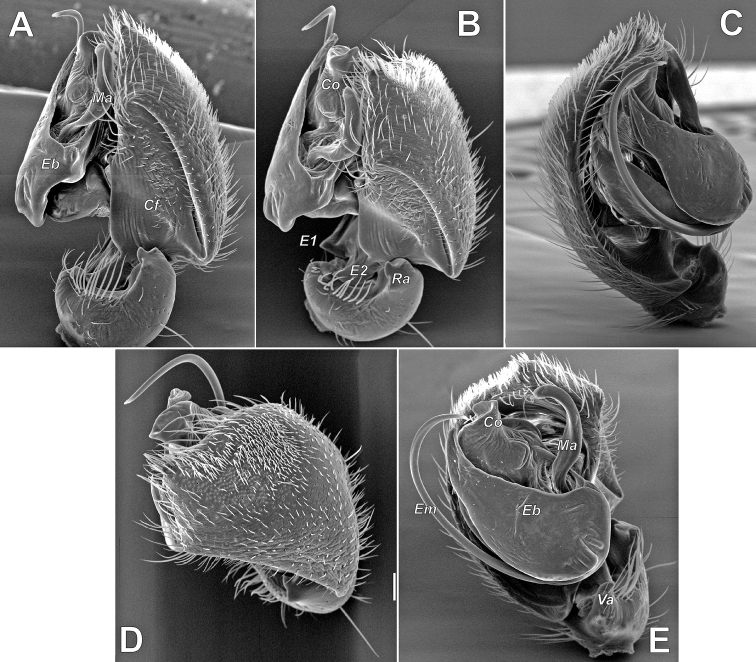
SEM images of the male palp of *Pax ellipita* sp. nov. **A** retrolateral **B** anteroretrolateral **C** proventral **D** posterodorsal **E** ventral. Abbreviations: *E1*, *E2* – pro- and retrolateral cymbial extensions, *Eb* – embolar base, *Em* – embolus, *Cf* – cymbial fold, *Co* – conductor, *Ma* – median apophysis, *Ra* – retrolateral apophysis, *Va* – ventral apophysis. Scale bar: 0.1 mm.

Figs 5A, B , 6 , 7 , 10A–E , 32

##### Type material.

***Holotype*** ♂ (MHNG), Iran: *Kermanshah Province*: north of Kermanshah, 34°28'N, 47°00'E, 18.06.1975 (A. Senglet).

##### Additional material.

Iran: *Lorestan Province*: 1♀ (NMP), Pol-e Tang, 60 km NW of Andimeshk, 32°51'N, 47°56'E, near the river Saimareh, 11.04.1977 (B. Pražan).

##### Etymology.

The specific epithet refers to Ellipi, an ancient kingdom located on the western side of the Zagros Mountains, between Babylonia at the west, Media at the north-east, Mannae at the north and Elam at the south.

##### Diagnosis.

The new species differs from *P. leila* sp. nov., the only other *Pax* species known from Iran, by the cymbium lacking a horn-like outgrowth (*vs.* present), by having a deep lateral fold of the cymbium (*vs.* lacking) and the epigyne with a rectangular median plate (*vs.* triangular). The male of *P. ellipita* sp. nov. differs from the rest of the species by having the RTA shorter than the tibia (*vs.* longer), while the female differs by the trilobate posterior margin of the epigyne (*vs.* solid) and copulatory openings located posteriorly (*vs.* anteriorly).

##### Description.

**Male.** Habitus as in Fig. 5B. Total length 5.07. Carapace 2.43 long, 1.27 wide at pars cephalica, 1.65 wide at pars thoracica. Eye sizes and interdistance of PMEs: AME: 0.11, ALE: 0.10, PME: 0.08, PLE: 0.10, PME–PME: 0.09. Carapace, sternum, labium, chelicerae and maxillae reddish brown, without any pattern. Chelicera with 2 promarginal teeth. Legs yellow, with few spines and without annulations. Abdomen dark brown, dorsally with large scutum covering almost the entire abdomen. Spinnerets pale, uniform in color. Measurements of legs: I: 5.49 (1.48, 0.62, 1.22, 1.10, 1.07), II: 4.88 (1.29, 0.60, 1.04, 1.05, 0.90), III: 4.74 (1.27, 0.61, 0.90, 1.19, 0.77), IV: 6.71 (1.80, 0.68, 1.39, 1.80, 1.04).

Palp as in Figs 6A–E, 7A–E. Femur slightly shorter than cymbium, 3.3 times longer than wide; patella globular; tibia wider than long with ventral apophysis (*Va*) and short RTA bifurcated at the tip (*Ra*); cymbium with large fold (*Cf*) and 2 extensions, pro- and retrolateral (*E1*, *E2*); bulb with large lamella, as long as bulb, tapering to the tip; median apophysis (*Ma*) long, about 5 times longer than wide; conductor (*Co*) heavily sclerotized; embolus (*Em*) long, originates at 6 o’clock position.

**Female.** Habitus as in Fig. 5A. Total length 5.33. Carapace 2.18 long, 1.08 wide at pars cephalica, 1.51 wide at pars thoracica. Eye sizes and interdistance of PMEs: AME: 0.06, ALE: 0.09, PME: 0.07, PLE: 0.09, PME–PME: 0.08. Coloration as in male, with lighter abdomen lacking a scutum. Measurements of legs: I: 5.36 (1.52, 0.60, 1.15, 1.09, 1.00), II: 4.76 (1.32, 0.61, 0.99, 1.04, 0.80), III: 4.53 (1.23, 0.60, 0.89, 1.11, 0.70), IV: 5.78 (1.42, 0.65, 1.38, 1.53, 0.80).

Epigyne as in Fig. 10A–E. Trilobate, wider than long; median plate rectangular (with subparallel lateral margins), ca. 3.6 times thinner than lateral lobe; receptacles oval, wider than long, touching each other.

##### Comments.

Because the female specimen was not collected together with the male, its assignment to this species is tentative and shall be confirmed when both sexes are collected together.

##### Distribution.

Known from the listed localities in Kermanshah and Lorestan provinces, western Iran (Fig. 32).

#### Pax leila sp. nov.

Taxon classificationAnimaliaAraneaeZodariidae

09834123-3293-57E4-9008-CCADEDB4806A

http://zoobank.org/9DEB48F2-08A4-482E-AEFF-405D9AAAA248

**Figure 8.**
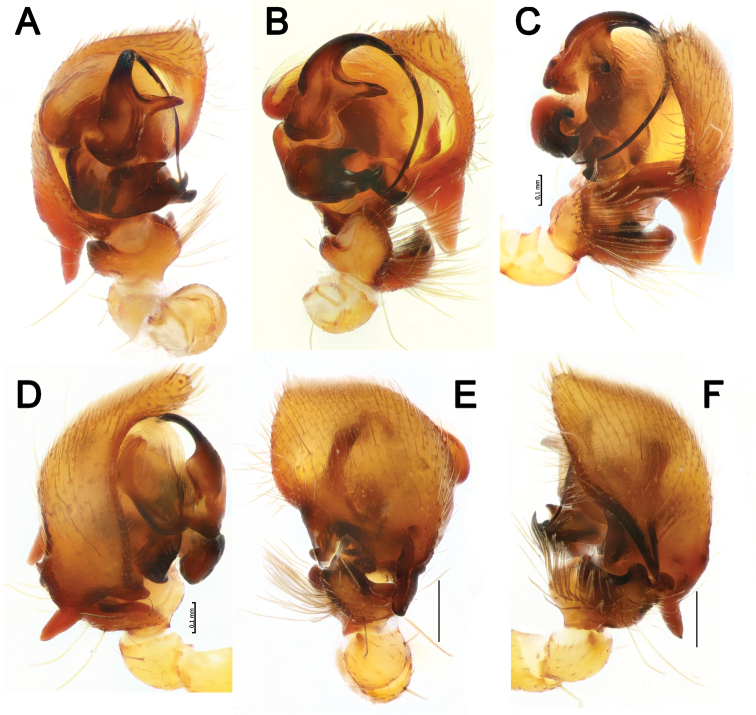
Male palp of *Pax leila* sp. nov. **A** proventral **B** ventral **C** ventroretrolateral **D** prolateral **E** dorsal **F** retrolateral. Scale bars: 0.2 mm, unless stated otherwise.

**Figure 9.**
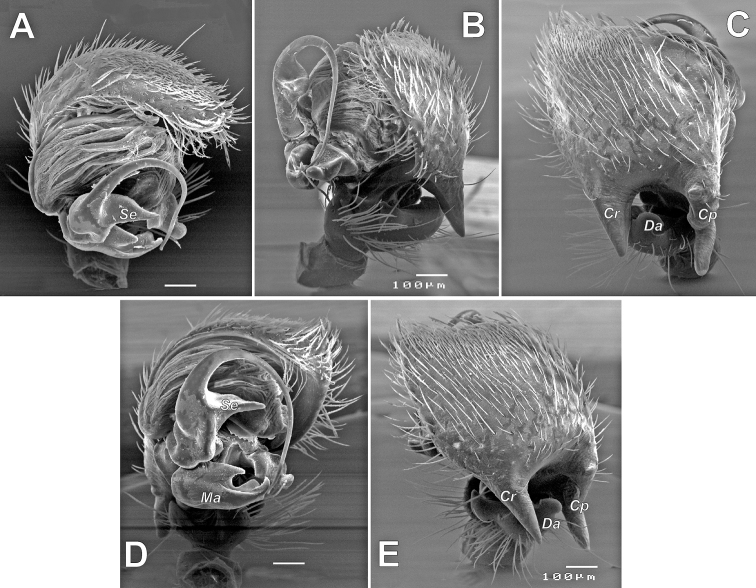
SEM images of the male palp of *Pax leila* sp. nov. **A** apicoventral **B** ventroretrolateral **C** prodorsal **D** ventral **E** retrodorsal. Abbreviations: *Cp* – prolateral cymbial process, *Cr* – retrolateral cymbial process, *Da* – dorsal apophysis, *Ma* – median apophysis, *Se* – spine of embolus base. Scale bars: 0.1 mm.

**Figure 10.**
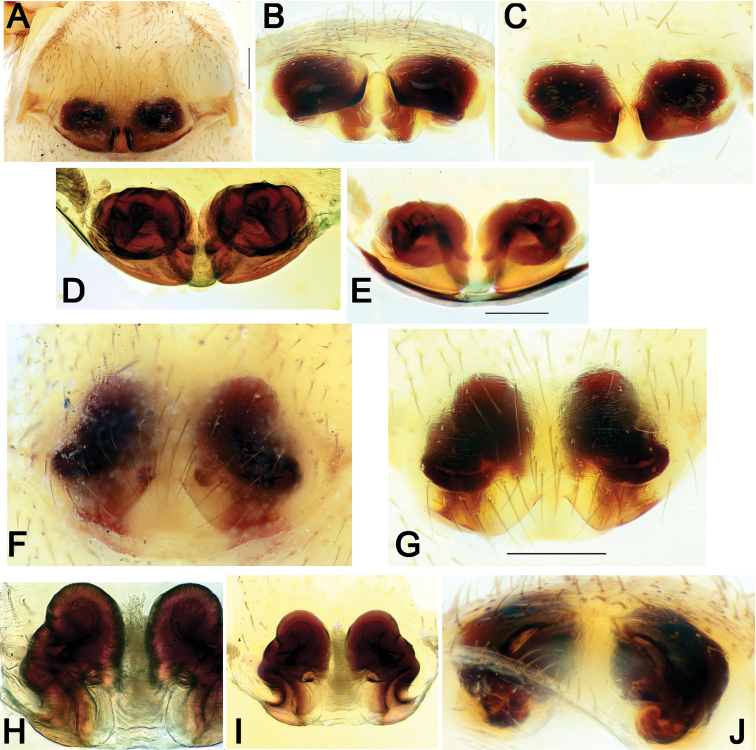
Epigynes of *Pax ellipita* sp. nov. (**A–E**) and *P. leila* sp. nov. (**F–J**) **A, F** intact, ventral **B, J** posterior **C, G** macerated, ventral **D, E, H, I** macerated, dorsal. Scale bars: 0.2 mm.

Figs 5C, D , 8 , 9 , 10F–J , 32

##### Type material.

***Holotype*** ♂ and ***paratype*** 1♀ (MHNG), Iran: *Fars Province*: road to Yasuj, 30°28'N, 51°30'E, 25.05.1974 (A. Senglet).

##### Etymology.

The specific epithet is a feminine given name in the Persian language, meaning “daughter of the night”. Noun in apposition.

##### Diagnosis.

The new species differs from all congeners by having a pair of spine-like cymbial outgrowths (Figs 8, 9C, E) (*vs.* lacking) and a triangular epigynal median plate (*vs.* absent or rectangular).

##### Description.

**Male.** Habitus as in Fig. 5C. Total length 4.42. Carapace 2.07 long, 1.03 wide at pars cephalica, 1.46 wide at pars thoracica. Eye sizes and interdistance of PMEs: AME: 0.09, ALE: 0.09, PME: 0.06, PLE: 0.09, PME–PME: 0.07. Carapace, sternum, labium, chelicerae and maxillae reddish brown, without any pattern. Chelicera with 2 promarginal teeth. Legs yellow, with few spines and without annulations. Abdomen dark brown, dorsally with large scutum covering 2/3 of the abdomen. Spinnerets pale, uniform in color. Measurements of legs: I: 4.51 (1.21, 0.50, 1.06, 0.93, 0.81), II: 3.89 (1.04, 0.52, 0.79, 0.83, 0.71), III: 3.62 (0.98, 0.50, 0.69, 0.85, 0.60), IV: 4.99 (1.33, 0.58, 1.05, 1.33, 0.70).

Palp as in Figs 8A–F, 9A–E. Tibia wider than long, with short apophysis; cymbium with 2 strong spine-like baso-posterior outgrowths (*Cp*, *Cr*), lateral fold lacking; embolus long, with robust triangular outgrowth near base (*Se*); median apophysis (*Ma*) very large, with 2 claw-like extensions; conductor small and strongly sclerotized.

**Female.** Habitus as in Fig. 5D. Total length 4.95. Carapace 1.97 long, 1.00 wide at pars cephalica, 1.31 wide at pars thoracica. Eye sizes and interdistance of PMEs: AME: 0.07, ALE: 0.08, PME: 0.06, PLE: 0.07, PME–PME: 0.08. Coloration as in male, with lighter abdomen lacking a scutum. Measurements of legs: I: 4.25 (1.21, 0.50, 0.99, 0.84, 0.71), II: 3.58 (0.94, 0.52, 0.74, 0.74, 0.64), III: 3.47 (0.90, 0.51, 0.70, 0.81, 0.55), IV: 4.71 (1.18, 0.53, 1.05, 1.21, 0.74).

Epigyne as in Fig. 10F–J. Epigyne trilobate, with triangular median plate, posteriorly as wide as lateral lobes; copulatory openings slit-like, broad; copulatory ducts wide, as wide as receptacles; receptacles globular, separated by less than their radii.

##### Comments.

Although this species is rather similar in general appearance to the generotype and the other species known from Iran, the bulb and cymbium conformation is very different from those of other species considered in *Pax* and most likely belongs to an undescribed genus.

##### Distribution.

Known only from the type locality in Fars Province, southwestern Iran (Fig. 32). This is the easternmost record of the genus *Pax*.

### Subfamily Zodariinae Thorell, 1881

#### Genus Acanthinozodium

Taxon classificationAnimaliaAraneaeZodariidae

Denis, 1966

AA4D61BB-05A7-5F32-99A8-14AB2E95E88E

##### Type species.

*Acanthinozodium spinulosum* Denis, 1966 from Libya.

##### Comments.

Twelve species are currently considered in this genus which are known from the Maghreb and adjacent countries in the south and in Socotra; previously, it was not recorded from Iran (WSC 2021). Although the male of the type species is unknown, *Acanthinozodium* comprises species having a unique gland located in the conical pit of the cymbium. The 11 species occurring in Iran and the one from Turkmenistan have the same conical pit and are thus placed in this genus; however, the male palpal tibia and particularly the bulbs are different from those of the species occurring in the Maghreb and surrounding countries. Further revisions are needed to clarify the taxonomy and composition of this group, which are beyond the scope of this paper.

#### Acanthinozodium atrisa sp. nov.

Taxon classificationAnimaliaAraneaeZodariidae

D5FCAC79-232E-53A3-9DAF-2BF008656B1E

http://zoobank.org/7BBBC164-8239-4F57-9F44-4F5DE93F8C24

**Figure 11.**
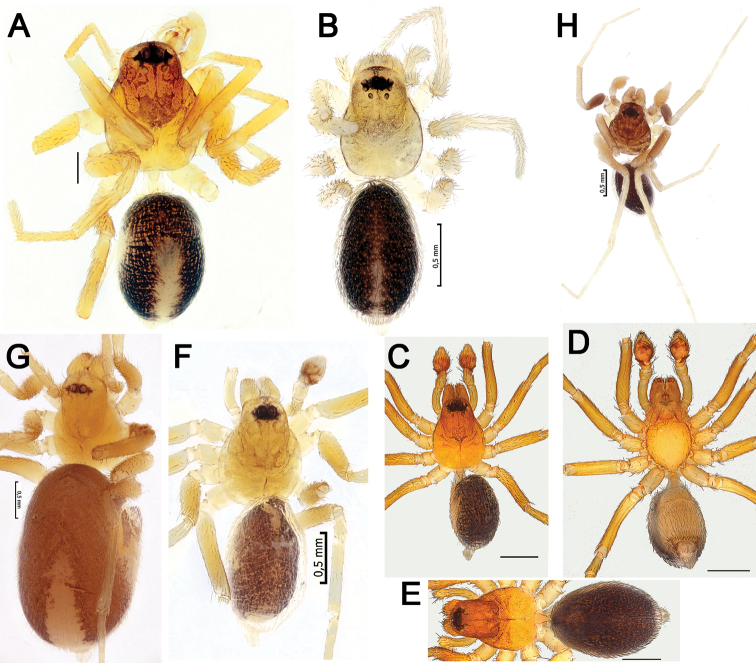
Habitus of *Acanthinozodium atrisa* sp. nov. (**A, B**) *A. niusha* sp. nov. (**C–E**), *A. parysatis* sp. nov. (**F, G**) and *A. sorani* sp. nov. (**H**) **A, C, F, H** males, dorsal **D** male, ventral **B, G, E** females, dorsal. Scale bars: 0.2 mm, unless stated otherwise.

**Figure 12.**
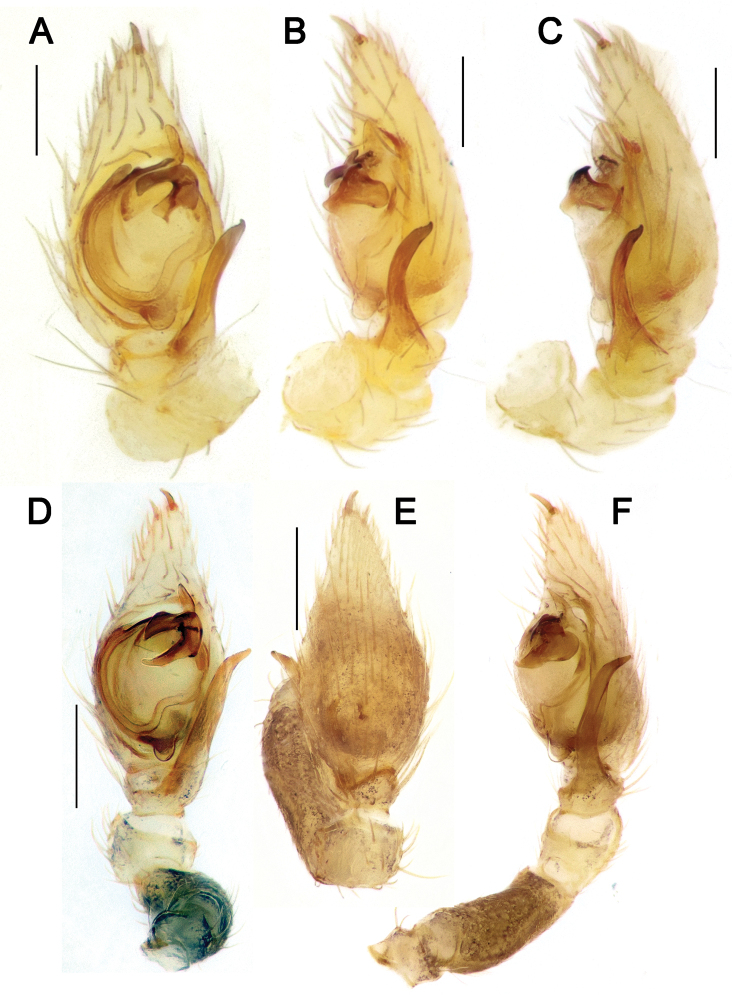
Male palps of *Acanthinozodium atrisa* sp. nov. (**A–C**) and *A. sorani* sp. nov. (**D–F**) **A, D** ventral **B, C, F** retrolateral **E** dorsal. Scale bars: 0.2 mm.

Figs 11A, B , 12A–C , 14A–C , 16A–D , 32

##### Type material.

***Holotype*** ♂ (MHNG), Iran: *Tehran Province*: Jamshidieh Mts., 35°49'N, 51°27'E, 05.2015 (A. Zamani). ***Paratypes***: 1♂2♀ (MHNG), same data as holotype; 5♂3♀ (MMUE), Latian Dam, 35°48'N, 51°08'E, 6–19.06.2000 (Y.M. Marusik); 1♂30♀1j. (MMUE), Plant Protection Institute, 35°40'N, 51°24'E, 7–22.06.2000 (Y.M. Marusik); 2♀ (MMUE), 5 km north of Tehran, Tochal Mts., 35°53'N, 51°20'E, 2000–2900 m, 16.06.2000 (Y.M. Marusik); 9♀1j. (ZMMU), 80 km east of Tehran, Damavand area, Aroo, 35°40'N, 52°27'E, 15.06.2000 (Y.M. Marusik & F. Mozaffarian); *Qazvin Province*: 1♂2♀ (MHNG), Agha Baba, 36°19'N, 49°49'E, 06.07.1974 (A. Senglet); 1♂1♀ (MHNG), Tarazan, Lowshan, 36°28'N, 49°31'E, 08.08.1974 (A. Senglet).

##### Etymology.

The specific epithet is a Persian feminine name meaning “queen of fire”. Noun in apposition.

##### Diagnosis.

The new species has an abdominal pattern similar to that of *A. parysatis* sp. nov. (dark abdomen with posterior median white stripe; less distinct in males) but differs by having a dark pars cephalica (*vs.* pale). The male palps of the two species differ by the RTA being thin and more than twice as long as the tibia in *A. atrisa* sp. nov. (*vs.* broad and as long as the tibia). Judging by the shape of the male palp, *A. atrisa* sp. nov. is probably closely related to *A. sorani* sp. nov., as both have a long RTA, a similarly shaped median apophysis, a posterior tegular process and an embolus with an anterior process. The two species differ by the shape of the embolic process (*Ep*), wider than the embolus and gently bent at the tip in *A. atrisa* sp. nov. (*vs.* spine-like). The epigyne of *A. atrisa* sp. nov. is most similar to that of *A. parysatis* sp. nov. in having a small anterior hood and a thin furrow between the lateral lobes but differs by the furrow being shorter than the bursa copulatrix (*vs.* the same length as bursa).

##### Description.

**Male** (holotype). Habitus as in Fig. 11A. Total length 1.78. Carapace 0.81 long, 0.39 wide at pars cephalica, 0.62 wide at pars thoracica. Eye sizes and interdistance of PMEs: AME: 0.07, ALE: 0.06, PME: 0.05, PLE: 0.05, PME–PME: 0.10. Carapace, sternum, labium, chelicerae and maxillae yellowish; carapace with irregular dark brown patterns. Chelicera with retromarginal tooth. Legs yellowish, without annulations. Abdomen dorsally black with longitudinal pale median band and grayish ventrally. Spinnerets pale, uniform in color. Measurements of legs: I: 2.39 (0.61, 0.25, 0.52, 0.53, 0.48), II: 2.07 (0.54, 0.26, 0.45, 0.46, 0.36), III: 2.06 (0.57, 0.25, 0.37, 0.48, 0.39), IV: 2.86 (0.69, 0.29, 0.64, 0.77, 0.47).

Palp as in Figs 12A–C, 14A–C. RTA (*Ra*) long (almost as long as bulb) and thin (7 times longer than wide), slightly bent; tegulum with posterior process, sperm duct almost straight retrolaterally, and gently bent along prolateral side; median apophysis wider than long; embolus (*Em*) broad basally, originating at about the 7 o’clock position, bifurcated terminally, with broad anterior process; embolus proper thin and straight, with small tubercle.

**Female.** Habitus as in Fig. 11B. Total length 2.38. Carapace 0.87 long, 0.39 wide at pars cephalica, 0.59 wide at pars thoracica. Eye sizes and interdistance of PMEs: AME: 0.07, ALE: 0.06, PME: 0.05, PLE: 0.05, PME–PME: 0.10. Coloration as in male, with paler carapace and less prominent abdominal median stripe. Measurements of legs: I: 2.36 (0.71, 0.24, 0.45, 0.51, 0.45), II: 2.18 (0.56, 0.28, 0.42, 0.48, 0.44), III: 2.16 (0.54, 0.27, 0.42, 0.51, 0.42), Fe IV: 0.76, rest of the segments missing.

Epigyne as in Fig. 16A–D. Fovea triangular, and small anterior hood present; bursae subtriangular, wider anteriorly, separated by less than one of their radii; receptacles smaller than bursae, ovoid, separated by more than 3 diameters.

##### Distribution.

Known only from the listed localities in Tehran and Qazvin provinces, northern Iran (Fig. 32).

#### Acanthinozodium niusha sp. nov.

Taxon classificationAnimaliaAraneaeZodariidae

93B02F86-D05D-51DA-8F61-A4DE2A2A7F92

http://zoobank.org/DA859B09-12D2-4370-A6B6-93C1BF274678

**Figure 13.**
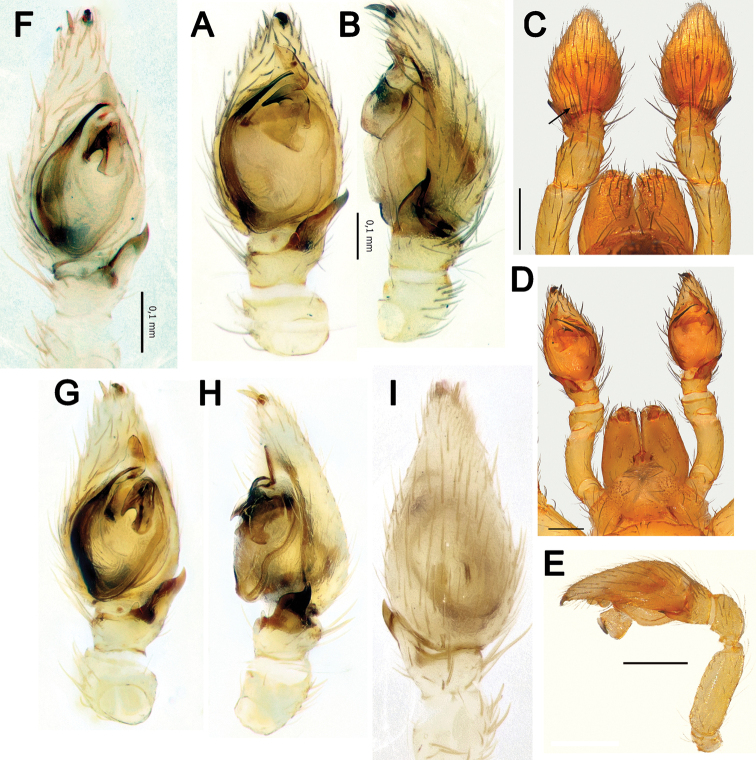
Male palps of *Acanthinozodium niusha* sp. nov. (**A–E**) and *A. parysatis* sp. nov. (**F–I**) **A, D, G, F** ventral **B, H** retrolateral **C, I** dorsal **E** dorsoretrolateral. Arrow on C pointing to cymbial groove. Scale bars: 0.2 mm, unless stated otherwise.

**Figure 14.**
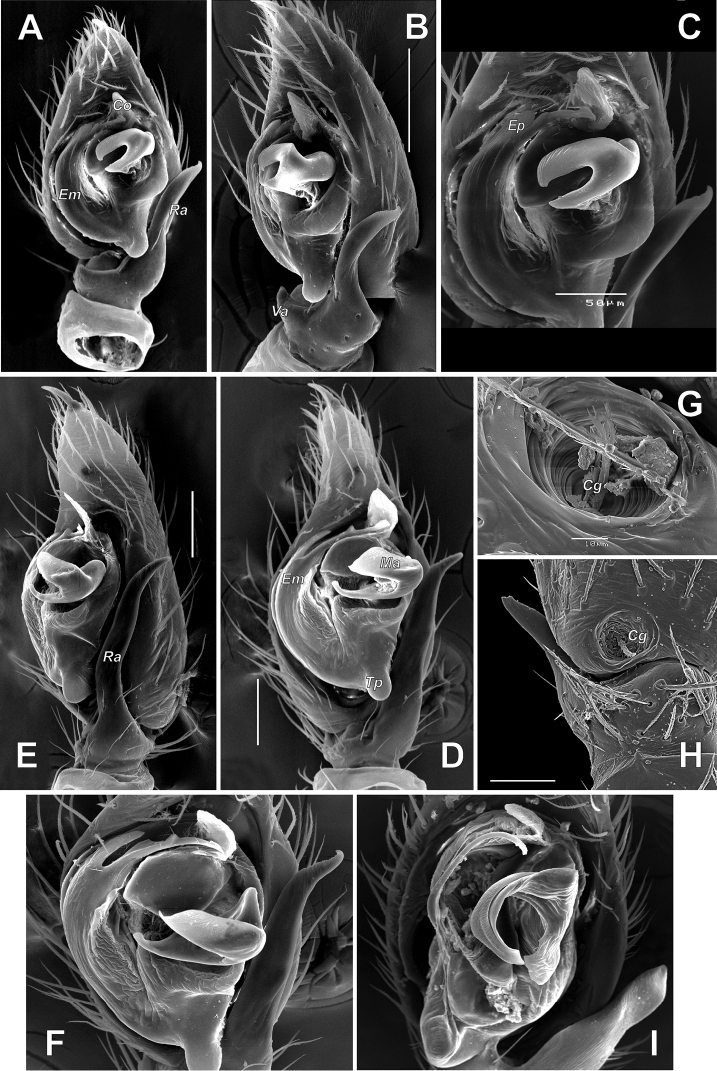
SEM images of the male palps of *Acanthinozodium atrisa* sp. nov. (**A–C**), *A. sorani* sp. nov. (**D–F**), *A. niusha* sp. nov. (**G, H**) and *A. dorsa* sp. nov. (**I**) **A, C, D, F, I** ventral **B, E** retrolateral **G, H** cymbial groove, dorsal. Abbreviations: *Cg* – cymbial groove, *Co* – conductor, *Em* – embolus, *Ep* – embolic process, *Ma* – median apophysis, *Ra* – retrolateral tibial apophysis, *Tp* – tegular process. Scale bars: 0.1 mm, unless stated otherwise.

**Figure 15.**
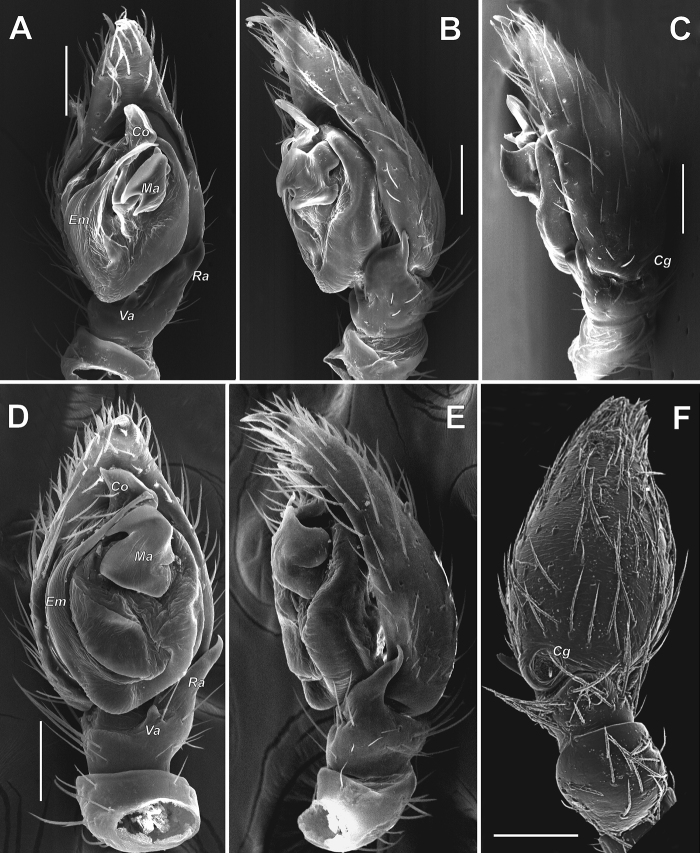
SEM images of the male palps of *Acanthinozodium parysatis* sp. nov. (**A–C**) and *A. niusha* sp. nov. (**D–F**) **A, D** ventral **B, E** retrolateral **C, F** dorsoretrolateral and dorsal. Abbreviations: *Cg* – cymbial groove, *Co* – conductor, *Em* – embolus, *Ma* – median apophysis, *Ra* – retrolateral tibial apophysis, *Va* – ventral apophysis. Scale bars: 0.1 mm.

Figs 11C–E , 13A–E , 14G, H , 15D–F , 17A–C , 32

##### Type material.

***Holotype*** ♂ (MHNG), Iran: *Markazi Province*: Shazand, 33°55'N, 49°24'E, 11.04.2015 (A. Zamani). ***Paratypes***: 5♂3♀ (MHNG), same data as holotype; *Isfahan Province*: 1♂1♀ (MHNG), Riz-e Landjan, 32°24'N, 51°19'E, 11.08.1973 (A. Senglet); *Fars Province*: 1♂1♀ (MHNG), Izad Khast, 31°31'N, 52°08'E, 12.06.1974 (A. Senglet).

##### Etymology.

The specific epithet is a Persian feminine name meaning “good listener”. Noun in apposition.

##### Diagnosis.

The male of the new species is most similar to *A. parysatis* sp. nov. from which it differs by a sharply tapering RTA, shorter tip of the cymbium (cf. Fig. 13A and 13F), the angle of embolus and the claw of the median apophysis. Female of *A. niusha* sp. nov. is similar to *A. atrisa* sp. nov. by having a similar anterior hood but differs by the longer furrow leading to the hood (as long as receptacle *vs.* 1.5 times shorter) and less separated receptacles.

##### Description.

**Male** (holotype). Habitus as in Fig. 11C, D. Total length 2.09. Carapace 0.94 long, 0.46 wide at pars cephalica, 0.69 wide at pars thoracica. Eye sizes and interdistance of PMEs: AME: 0.10, ALE: 0.07, PME: 0.06, PLE: 0.06, PME–PME: 0.12. Carapace, sternum, labium, chelicerae and maxillae yellowish; carapace with irregular dark patterns. Chelicera with one retromarginal tooth. Legs yellowish, without annulations. Abdomen dorsally black, grayish ventrally. Spinnerets pale, uniform in color. Measurements of legs: I: 1.67+missing tarsus (0.74, 0.31, 0.62, missing), II: 2.56 (0.66, 0.30, 0.52, 0.59, 0.49), III: 2.43 (0.60, 0.33, 0.46, 0.59, 0.45), IV: 3.43 (0.90, 0.31, 0.81, 0.89, 0.52).

Palp as in Figs 13A–E, 14G, H, 15D–F. RTA (*Ra*) as long as wide and as long as tibia, basal part very wide, sharply tapering, tip bent ventrally; cymbium 1.5 times longer than wide; posterior part of tegulum with broad and indistinct projection postero-prolaterally; median apophysis (*Ma*) with massive base, much larger than the claw of median apophysis; sperm duct gradually tracking along margin of tegulum; tip of embolus (*Em*) straight and lacking any processes or tubercles.

**Figure 16.**
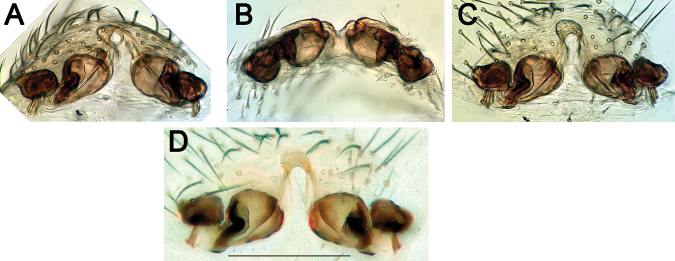
Epigyne of *Acanthinozodium atrisa* sp. nov. **A** posteroventral **B** posterior **C, D** ventral. Scale bar: 0.2 mm.

**Female.** Habitus as in Fig. 11E. Total length 2.10. Carapace 0.86 long, 0.44 wide at pars cephalica, 0.69 wide at pars thoracica. Eye sizes and interdistance of PMEs: AME: 0.07, ALE: 0.06, PME: 0.05, PLE: 0.05, PME–PME: 0.10. Coloration as in male. Measurements of legs: I: 2.68 (0.73, 0.30, 0.59, 0.61, 0.45), II: 2.58 (0.74, 0.24, 0.53, 0.58, 0.49), III: 2.32 (0.59, 0.29, 0.50, 0.55, 0.39), IV: 3.45 (0.89, 0.33, 0.80, 0.92, 0.51).

**Figure 17.**
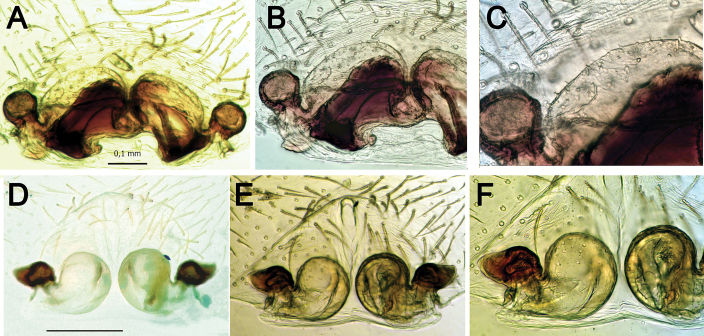
Ventral view of epigynes of *Acanthinozodium niusha* sp. nov. (**A–C**) and *A. parysatis* sp. nov. (**D–F**). Scale bar: 0.2 mm, unless stated otherwise.

Epigyne as in Fig. 17A–C. Fovea about 3 times wider than long; hood as wide as fovea, bursae ovoid, weakly sclerotized, with fine pores (Fig. 17C); receptacles round, spaced by over 5 diameters.

##### Distribution.

Known only from the listed localities in Fars, Isfahan and Markazi provinces, central Iran (Fig. 32).

#### Acanthinozodium ovtchinnikovi sp. nov.

Taxon classificationAnimaliaAraneaeZodariidae

3027AB94-46A6-5E6D-A6A6-4FCE757D5270

http://zoobank.org/DB25B91A-E827-4482-A2D4-1EC1D5012BF2

Figs 22D, E, G–I , 32

##### Type material.

***Holotype*** ♂ palp (ZMMU), Turkmenistan: *Mary Region*: Kushka Dist., ca 18 km S of Kyzyldzhar Kordon, ca 1 km ESE of Eroilandaz, 35°39'N, 61°50'E, 7.04.2002 (A.V. Gromov).

##### Etymology.

The new species is named after our late colleague Sergei V. Ovtchinnikov who made important contributions to the study of Central Asian spiders.

##### Diagnosis.

The new species differs from the congeners in the region (except *A. parmida* sp. nov.) by the small size of the palp (cymbium 0.28 long, *vs.* > 0.6) and the unique embolus which has a long furrow with a serrate ventral margin (Fig. 22E). It is closely related to *A. parmida* sp. nov. from central Iran, from which it can be differentiated by having a longer than wide bulb (*vs.* almost as long as wide), relatively longer cymbium (length/width ratio 1.86 vs. 1.4), and a different shape of median apophysis (anterior portion larger than posterior one, *vs.* opposite; cf. Figs 22G and 19B).

##### Description.

**Male.** Body missing. It is assumed to be a very small zodariid based on the size of the palp.

Palp as in Fig. 22D, E, G–I. Ventral tibial apophysis lacking; RTA almost triangular, slightly longer than tibia; cymbium longer than wide; sperm duct tracking margin of tegulum, lacking any turns; median apophysis longer than wide, with anterior part larger than posterior part; embolus originates at about 8:00 o'clock position with its terminal 2/3 having a longitudinal furrow (*Er*) with a finely serrated ventral margin.

**Female.** Unknown.

##### Note.

The sample collected by Gromov contained three males. The palp of one specimen was dissected and imaged with a SEM in 2005. Then, all three specimens were given to Ovtchinnikov who was planning to revise this group. After his death, his collection was transferred to Almaty, Kazakhstan by Alexander Gromov and is now inaccessible.

##### Distribution.

Known only from the type locality in Mary Region, southeastern Turkmenistan (Fig. 32).

#### Acanthinozodium parysatis sp. nov.

Taxon classificationAnimaliaAraneaeZodariidae

440E5E09-922D-5E9C-8DE0-0B36BC3DA4CB

http://zoobank.org/92A3E44F-1EC2-4246-A21E-CAEED957E5BE

Figs 11F, G , 13F–I , 15A–C , 17D–F , 32

##### Type material.

***Holotype*** ♂ (MHNG), Iran: *Ardabil Province*: Kivi Pain, 37°41'N, 48°21'E, 09.06.1975 (A. Senglet). ***Paratypes***: 1♂ (MHNG), same data as holotype; 3♀1♂ palp (MHNG), Iran: *Qazvin Province*: Shahrak, 36°25'N, 50°30'E, 02.07.1975 (A. Senglet).

##### Etymology.

The specific epithet is an ancient Persian feminine name, meaning “fairy-like”. Noun in apposition.

##### Diagnosis.

The male palp of *A. parysatis* sp. nov. is similar to that of *A. niusha* sp. nov. by the overall shape of the median apophysis and RTA but differs in lacking a retrolateral extension on the median apophysis (*vs.* present) and by having a shorter, stouter RTA (*vs.* longer and tapering). The epigyne of the new species is most similar to that of *A. atrisa* sp. nov. in having a small anterior hood and thin furrow between the lateral lobes but differs by the relative length of the anterior part of the fovea being shorter than the bursae in *A. parysatis* sp. nov. (*vs.* as long as bursae).

##### Description.

**Male** (holotype). Habitus as in Fig. 11F. Total length 2.23. Carapace 1.03 long, 0.46 wide at pars cephalica, 0.80 wide at pars thoracica. Eye sizes and interdistance of PMEs: AME: 0.10, ALE: 0.07, PME: 0.06, PLE: 0.05, PME–PME: 0.10. Carapace, sternum, labium, chelicerae and maxillae yellowish. Legs yellowish, slightly darker at femora, without annulations. Abdomen dorsally grayish, pale ventrally. Spinnerets pale, uniform in color. Measurements of legs: I: 2.98+missing tarsus (0.95, 0.37, 0.80, 0.86, missing), II: 3.13 (0.77, 0.34, 0.66, 0.79, 0.57), III: 3.03 (0.76, 0.35, 0.58, 0.86, 0.48), IV: 3.44 (1.00, 0.30, 0.73, 0.85, 0.56).

Palp as in Figs 13F–I, 15A–C. RTA (*Ra*) relatively short and stout, with a finger-like projection dorsally; tegulum with posterior process, sperm duct almost straight retrolaterally, and gently bent along prolateral side; median apophysis (*Ma*) wider than long; embolus broad basally, originating at about the 7 o’clock position; embolus proper thin, slightly twisted apically.

**Female.** Habitus as in Fig. 11G. Total length 3.68. Carapace 1.34 long, 0.60 wide at pars cephalica, 0.94 wide at pars thoracica. Eye sizes and interdistance of PMEs: AME: 0.09, ALE: 0.08, PME: 0.07, PLE: 0.07, PME–PME: 0.16. Coloration generally as in male. Abdomen dorsally with a pale median band occupying half of the abdomen’s length. Measurements of legs: I: 3.79 (0.90, 0.40, 0.84, 1.01, 0.64), II: 2.90+missing tarsus (0.94, 0.36, 0.74, 0.86, missing), III: 3.48 (0.88, 0.40, 0.65, 1.03, 0.52), IV: 5.09 (1.33, 0.48, 1.13, 1.47, 0.68).

Epigyne as in Fig. 17D–F. Fovea triangular and small anterior hood present; bursae round, separated by less than one of their radii; receptacles smaller than bursae, triangular, separated by more than 3 diameters.

##### Distribution.

Known only from the listed localities in Ardabil and Qazvin provinces, northern and northwestern Iran (Fig. 32).

#### Acanthinozodium sorani sp. nov.

Taxon classificationAnimaliaAraneaeZodariidae

A5D28975-2A41-5787-BF60-F44464C2C3A0

http://zoobank.org/CD502D82-8DBC-4E66-9626-D9CD571D1DAC

Figs 11H , 12D–F , 14D–F , 32

##### Type material.

***Holotype*** ♂ (MHNG), Iran: *Kurdistan Province*: Santeh, 36°11'N, 46°32'E, 23.06.1975 (A. Senglet). ***Paratypes***: 6♂ (MHNG), Marivan, 5.2017 (A. Zamani); *East Azerbaijan Province*: 2♂ (MHNG), north of Bonati, 37°26'N, 45°57'E, 04.06.1975 (A. Senglet).

##### Etymology.

The specific epithet refers to a dialect or a language of the Kurdish languages that is spoken in Iraq, mainly in Iraqi Kurdistan, as well as the Kurdistan Province, Kermanshah Province, and West Azerbaijan Province of western Iran. Noun in apposition.

##### Diagnosis.

The new species is most similar to *A. atrisa* sp. nov. but differs by embolic anterior process which is spine-like and thinner than the embolus proper in *A. sorani* sp. nov. (*vs.* broad) and also by the relative length of the RTA, longer than bulb in *A. sorani* sp. nov. (*vs.* shorter).

##### Description.

**Male** (holotype). Habitus as in Fig. 11H. Total length 2.11. Carapace 1.00 long, 0.46 wide at pars cephalica, 0.72 wide at pars thoracica. Eye sizes and interdistance of PMEs: AME: 0.09, ALE: 0.07, PME: 0.06, PLE: 0.06, PME–PME: 0.12. Carapace, sternum, labium, chelicerae and maxillae yellowish brown; carapace with irregular dark patterns. Chelicera with one retromarginal tooth. Legs yellowish, dark brown at femora I and II, without annulations. Abdomen dorsally black, grayish ventrally. Spinnerets pale, uniform in color. Measurements of legs: I: 3.87 (0.97, 0.37, 0.89, 0.91, 0.64), II: 3.47 (0.92, 0.32, 0.72, 0.88, 0.63), Fe III: 0.88, other segments missing, IV: 4.51 (1.06, 0.36, 1.12, 1.33, 0.64).

Palp as in Figs 12D–F, 14D–F. RTA (*Ra*) long (longer than bulb) and thin (about 6 times longer than wide), slightly bent; tegulum with posterior process (*Tp*), sperm duct almost straight retrolaterally, and gently bent along prolateral side; median apophysis (*Ma*) wider than long; embolus (*Em*) broad basally, bifurcated terminally, with spine-like anterior process; embolus proper thin and straight, with small tubercle.

**Female.** Unknown.

##### Distribution.

Known only from the listed localities in Kurdistan and East Azerbaijan provinces, western and northwestern Iran (Fig. 32).

#### Acanthinozodium armita sp. nov.

Taxon classificationAnimaliaAraneaeZodariidae

E852B3FA-E0A6-57EC-B544-8E7C4B62F79F

http://zoobank.org/BCEEC5DD-E5BA-483A-A43F-DF518429CC54

**Figure 18.**
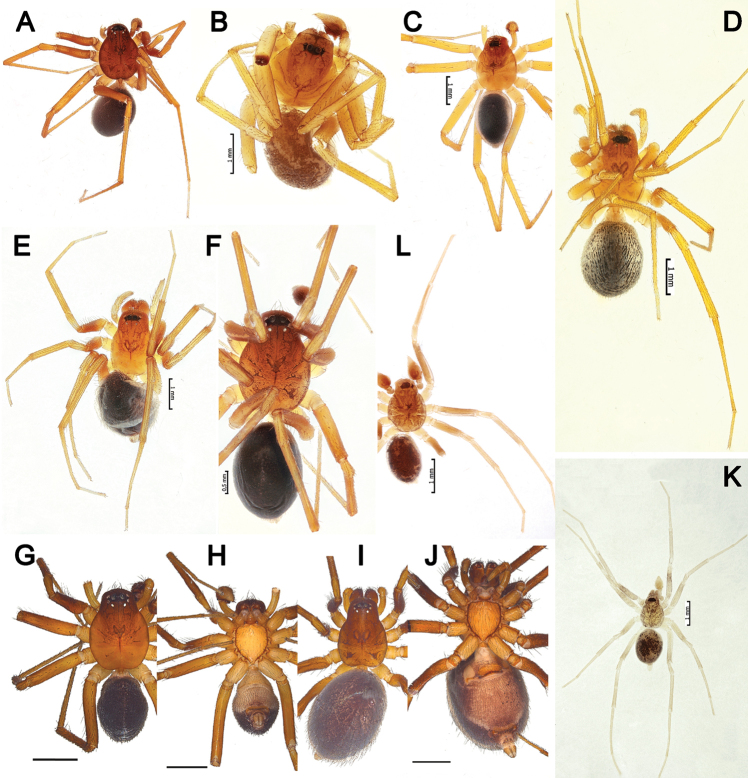
Habitus of *Acanthinozodium armita* sp. nov. (**A**), *A. diara* sp. nov. (**B**), *A. dorsa* sp. nov. (**C, D**), *A. elburzicum* sp. nov. (**E–J**), *A. kiana* sp. nov. (**K**) and *A. masa* sp. nov. (**L**) **A–C, F, G, K, L** males, dorsal **H** male, ventral **D, E, I** females, dorsal **J** female, ventral. Scale bars: 0.2 mm, unless stated otherwise.

Figs 18A , 20A–C , 22A–C, F , 33

##### Type material.

***Holotype*** ♂ (MMUE), Iran: *Tehran Province*: northwest of Tehran, Sardor area, 35°50'N, 51°05'E, 13.06.2000 (Y.M. Marusik). ***Paratype*** ♂ (NHMW), Iran: Haji Abad, 06.1972 (G. Pretzman & A. Konetschnig).

##### Etymology.

The specific epithet is a Persian feminine name meaning “righteous”, “virtuous”, and “good”. Noun in apposition.

##### Diagnosis.

The new species differs from all congeners in the region by the short RTA having 2 claw-like outgrowths on the tip (*vs.* tip tapering and lacking 2 claws) and also by the modified embolus, widening near the tip (*vs.* unmodified).

##### Description.

**Male** (holotype). Habitus as in Fig. 18A. Total length 4.70. Carapace 2.18 long, 0.92 wide at pars cephalica, 1.52 wide at pars thoracica. Eye sizes and interdistance of PMEs: AME: 0.19, ALE: 0.12, PME: 0.10, PLE: 0.12, PME–PME: 0.25. Carapace dark brown, with irregular dark patches and lines. Sternum, labium and maxillae light brown. Chelicera dark brown, with one tooth. Legs light brown, without annulations. Abdomen black, without any pattern; slightly lighter ventrally. Spinnerets pale, uniform in color. Measurements of legs: I: 9.49 (2.38, 0.71, 2.16, 2.76, 1.48), II: 8.76 (2.10, 0.78, 1.98, 2.50, 1.40), III: 8.36 (2.11, 0.70, 1.69, 2.73, 1.13), IV: 11.42 (3.00, 0.81, 2.60, 3.59, 1.42).

**Figure 19.**
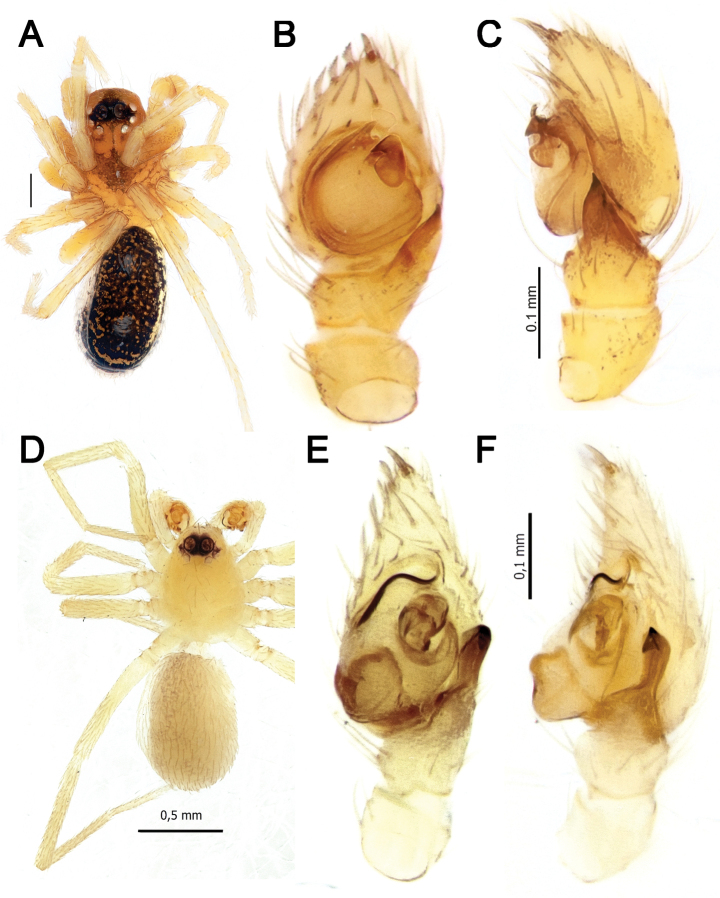
Male habitus and palps of *Acanthinozodium parmida* sp. nov. (**A–C**) and *Zodarion expers* (**D–F**) **A, D** habitus, dorsal **B, E** palp, ventral **C, F** palp, retrolateral. Scale bar: 0.2 mm (**A**).

**Figure 20.**
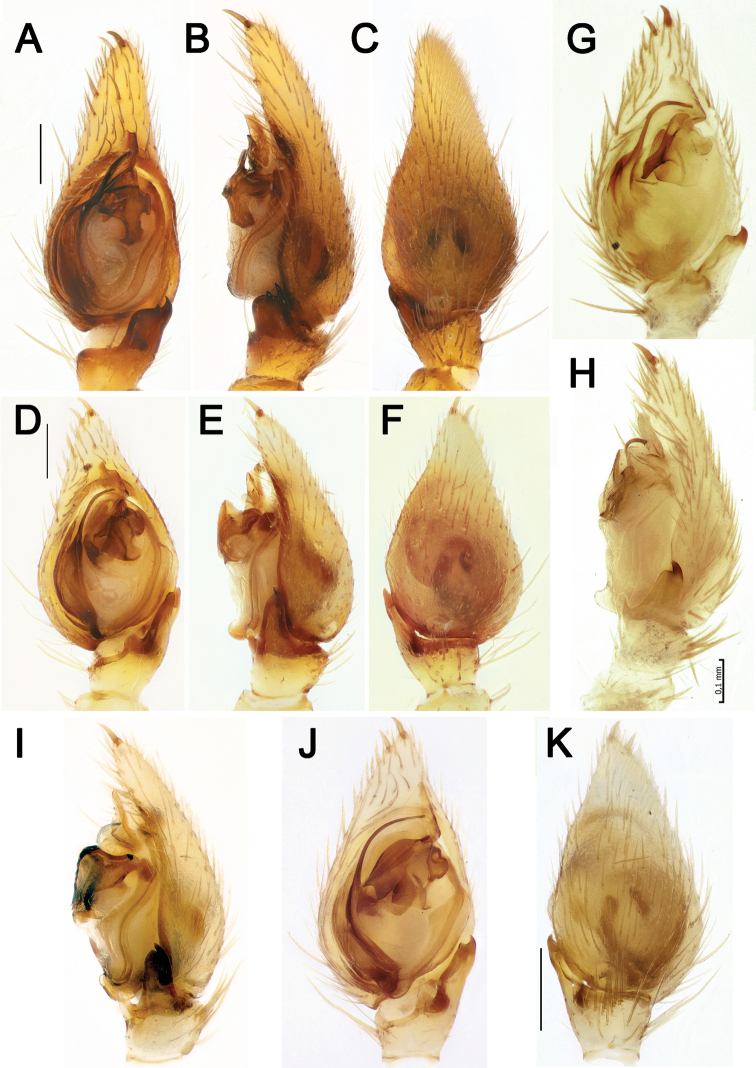
Male palps of *Acanthinozodium armita* sp. nov. (**A–C**), *A. diara* sp. nov. (**D–F**), *A. kiana* sp. nov. (**G, H**) and *A. masa* sp. nov. (**I–K**) **A, D, G, J** ventral **B, E, H, I** retrolateral **C, F, K** dorsal. Scale bars: 0.2 mm, unless stated otherwise.

Palp as in Figs 20A–C, 22A–C, F. RTA short and stout, with 2 claw-like outgrowths on the tip; ventral apophysis absent; tegulum longer than wide; median apophysis (*Ma*) longer than wide, with outgrowths on all four sides; embolus with a distinct ridge (*Er*); embolus proper twisted basally and widened near the tip.

**Female.** Unknown.

##### Distribution.

Known from the type locality in Tehran Province, northern Iran, and another locality (Haji Abad) which cannot be georeferenced because there are many places with this name in Iran (Fig. 33).

#### Acanthinozodium diara sp. nov.

Taxon classificationAnimaliaAraneaeZodariidae

C61523CF-4B07-574D-A528-FA84A2573392

http://zoobank.org/AFBD4904-D586-4FD5-A261-EC34D20EB469

Figs 18B , 20D–F , 23D–F , 33

##### Type material.

***Holotype*** ♂ (MHNG), Iran: *Ilam Province*: Dizgaran, 33°44'N, 46°59'E, 16.5.1974 (A. Senglet). ***Paratype*** ♂ (MMUE), IRAN: *Lorestan Province*: Dorood, 31.7.2011 (S. Zaruni).

##### Etymology.

The specific epithet is a Persian feminine name meaning “motherland”. Noun in apposition.

##### Diagnosis.

*Acanthinozodium diara* sp. nov. is very similar to *A. masa* sp. nov. by the shape of the RTA, the ventral tibial apophysis and the curvature of the embolus but differs by the shape of the median apophysis (cf. Fig. 20D and 20J) and the conductor having a small retrolateral indentation apically (*vs.* without indentation).

##### Description.

**Male** (holotype). Habitus as in Fig. 18B. Total length 4.70. Carapace 2.18 long, 0.92 wide at pars cephalica, 1.52 wide at pars thoracica. Eye sizes and interdistance of PMEs: AME: 0.19, ALE: 0.12, PME: 0.10, PLE: 0.12, PME–PME: 0.25. Carapace dark brown, with irregular dark patches and lines. Sternum, labium and maxillae light brown. Chelicera dark brown, with one retromarginal tooth. Legs light brown, without annulations. Abdomen black, without any pattern; slightly lighter ventrally. Spinnerets pale, uniform in color. Measurements of legs: I: 9.49 (2.38, 0.71, 2.16, 2.76, 1.48), II: 8.76 (2.10, 0.78, 1.98, 2.50, 1.40), III: 8.36 (2.11, 0.70, 1.69, 2.73, 1.13), IV: 11.42 (3.00, 0.81, 2.60, 3.59, 1.42).

Palp as in Figs 20D–F, 23D–F. RTA long and conical, with a small projection apically (Fig. 20E); tegulum with posterior process; ventral apophysis small and conical; median apophysis almost as long as wide, with outgrowths on all four sides, posterior one largest; embolus broad basally, originating at about the 7 o’clock position; embolus proper thin and steadily curving.

**Female.** Unknown.

##### Distribution.

Known from the listed localities in Ilam and Lorestan provinces, western Iran (Fig. 33).

#### Acanthinozodium dorsa sp. nov.

Taxon classificationAnimaliaAraneaeZodariidae

A34DB4A2-49F3-5C48-A7A6-70818BA31422

http://zoobank.org/CDA6AFFA-3C76-4F74-8BC4-03B5DF32B011

**Figure 21.**
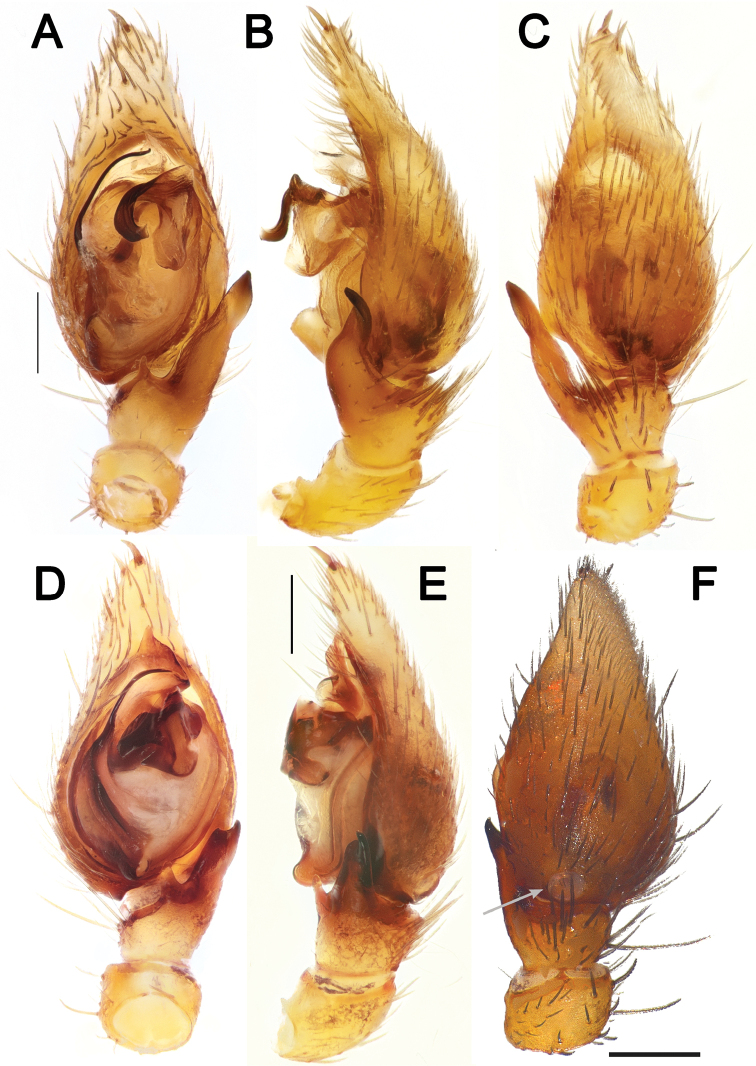
Male palps of *Acanthinozodium dorsa* sp. nov. (**A–C**) and *A. elburzicum* sp. nov. (**D–F**) **A, D** ventral **B, E** retrolateral **C, F** dorsal, with arrow pointing to cymbial groove. Scale bars: 0.2 mm.

Figs 14I , 18C, D , 21A–C , 24C, D , 30F–I , 33

##### Type material.

***Holotype*** ♂ and ***paratypes*** 26♂16♀ (MMUE), Iran: *Fars Province*: 50 km NE of Shiraz, Bamoo reserve area, 29°45'N, 52°45'E, 18–28.05.2000 (Y.M. Marusik).

##### Etymology.

The specific epithet is a Persian feminine name meaning “precious”. Noun in apposition.

##### Diagnosis.

The male of the new species differs from all congeners in the region by the prolateral outgrowth of the median apophysis projecting ventrally (*vs.* not projecting) and the long and broad RTA, apically twisted and lacking outgrowths (*vs.* with 1–2 outgrowths). The female is most similar to *A. elburzicum* sp. nov. but differs by the epigynal hood being longer than wide (*vs.* wider than long) and less separated receptacles (ca. 2.5 times of their diameter *vs.* 4).

##### Description.

**Male** (holotype). Habitus as in Fig. 18C. Total length 3.85. Carapace 1.87 long, 0.89 wide at pars cephalica, 1.39 wide at pars thoracica. Eye sizes and interdistance of PMEs: AME: 0.15, ALE: 0.12, PME: 0.09, PLE: 0.09, PME–PME: 0.20. Carapace yellowish brown, darker at pars cephalica. Sternum, labium, maxillae and chelicerae light brown. Legs yellowish, without annulations. Abdomen dark brown dorsally, pale ventrally. Spinnerets pale, uniform in color. Measurements of legs: I: 8.20 (2.02, 0.68, 1.90, 2.19, 1.41), II: 6.89 (1.68, 0.68, 1.49, 1.99, 1.05), III: 7.23 (1.88, 0.64, 1.45, 2.13, 1.13), IV: 8.71 (2.18, 0.62, 2.11, 2.70, 1.10).

Palp as in Figs 14I, 21A–C, 24C, D. RTA long and broad, almost as long as the bulb, twisting ventrally toward the apex; ventral apophysis small and finger shaped; tegulum with posterior process; median apophysis (*Ma*) with a distinct prolateral projection, winding ventrally to a blunt tip; embolus (*Em*) originating at about the 8:30 o’clock position; embolus proper thin and slightly curving near the base and apex.

**Female.** Habitus as in Fig. 18D. Total length 4.46. Carapace 1.93 long, 0.96 wide at pars cephalica, 1.46 wide at pars thoracica. Eye sizes and interdistance of PMEs: AME: 0.15, ALE: 0.12, PME: 0.09, PLE: 0.10, PME–PME: 0.15. Coloration as in male. Measurements of legs: I: 6.58 (1.39, 0.56, 1.52, 1.88, 1.23), II: 6.49 (1.62, 0.69, 1.34, 1.77, 1.07), III: 6.24 (1.71, 0.63, 1.25, 1.85, 0.80), IV: 8.45 (2.24, 0.72, 1.90, 2.60, 0.99).

Epigyne as in Fig. 30F–I. Epigynal plate over 3 times wider than long; anterior hood ca. 2 times longer than wide; receptacles subrectangular, separated by about 2.5 times of their widths.

##### Distribution.

Known from the type locality in Fars Province, southern Iran (Fig. 33).

#### Acanthinozodium elburzicum sp. nov.

Taxon classificationAnimaliaAraneaeZodariidae

46CFFF9D-FBBB-518D-9F98-02AC3F372F3E

http://zoobank.org/61538C61-2E8A-4F21-8289-B9129E5E4965

Figs 18E–J , 21D–F , 23A–C , 26E , 29A , 30A–E , 33

##### Type material.

***Holotype*** ♂ (MHNG), Iran: *Tehran Province*: Jamshidieh Mts., 35°49'N, 51°27'E, 05.2015 (A. Zamani). ***Paratypes***: 1♀ (MHNG), Pardisan Park, 35°44'N, 51°21'E, 05.2015 (A. Zamani); 5♂2♀1j. (MMUE), Latian Dam, 35°48'N, 51°08'E, 06–19.06.2000 (Y.M. Marusik); 3♂1♀ (MMUE), Plant Protection Institute, 35°40'N, 51°24'E, 07–22.06.2000 (Y.M. Marusik).

##### Etymology.

The specific epithet refers to the Elburz Mountain Range, in which the type locality of the new species is situated.

##### Diagnosis.

The male of the new species is similar to *A. diara* sp. nov. by the similar shape of the RTA and they both have a small retrolateral indentation apically on the conductor, but differs by the shape of the median apophysis, which is with a distinctly larger prolateral outgrowth. The female of the new species is most similar to *A. dorsa* sp. nov. but differs by the epigynal hood being wider than long (*vs.* longer than wide) and receptacles separated by about 4 diameters (*vs.* ca. 2.5).

##### Description.

**Male** (holotype). Habitus as in Fig. 18F–H (holotype: 18F). Total length 4.30. Carapace 2.08 long, 0.97 wide at pars cephalica, 1.49 wide at pars thoracica. Eye sizes and interdistance of PMEs: AME: 0.14, ALE: 0.12, PME: 0.08, PLE: 0.09, PME–PME: 0.15. Carapace dark brown, lighter in pars cephalica, with irregular dark patches and lines. Sternum, labium and maxillae light brown. Chelicera (Fig. 26E) dark brown, with one retromarginal tooth. Legs slightly lighter than carapace, without annulations. Abdomen dorsally black and with large scutum; ventrally grayish, without any pattern. Spinnerets pale, uniform in color. Measurements of legs: I: 7.52 (1.79, 0.71, 1.75, 2.03, 1.24), II: 6.79 (1.87, 0.69, 1.40, 1.83, 1.00), III: 6.90 (1.78, 0.74, 1.40, 2.06, 0.92), IV: 9.19 (2.48, 0.83, 2.15, 2.84, 0.89).

Palp as in Figs 21D–F, 23A–C, 29A. RTA (*Ra*) long and conical, with a small projection apically; ventral apophysis (*Va*) small and conical; tegulum with posterior process; median apophysis (*Ma*) wider than long, with outgrowths on all four sides, the triangular prolateral one the largest; embolus (*Em*) originating at about the 7 o’clock position; embolus proper thin and steadily curving.

**Figure 22.**
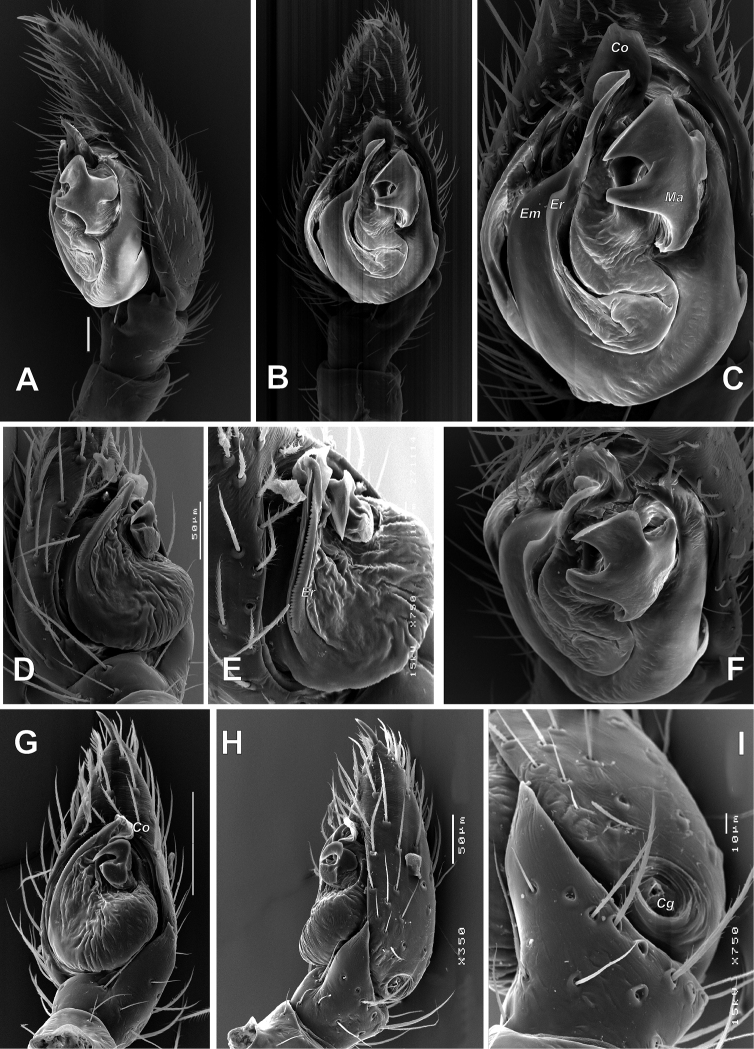
SEM images of the male palps of *Acanthinozodium armita* sp. nov. (**A–C, F**) and *A. ovtchinnikovi* sp. nov. (**D, E, G–I**) **A, H** retroventral and retrolateral **B, C, G** ventral **D–F** proventral, apicoproventral and apicoventral **I** retrolateral tibial and cymbial groove. Abbreviations: *Cg* – cymbial groove, *Co* – conductor, *Em* – embolus, *Er* –embolar ridge, *Ma* – median apophysis. Scale bars: 0.1 mm, unless stated otherwise.

**Figure 23.**
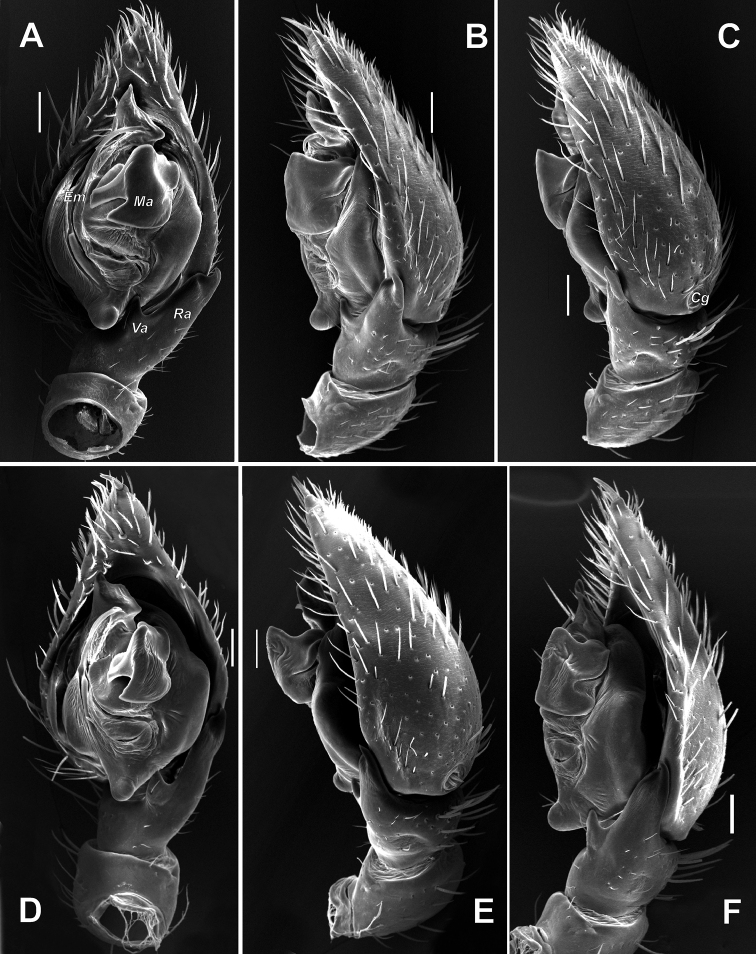
SEM images of the male palps of *Acanthinozodium elburzicum* sp. nov. (**A–C**) and *A. diara* sp. nov. (**D–F**) **A, D** ventral **B, F** retrolateral **C, E** dorsoretrolateral. Abbreviations: *Cg* – cymbial groove, *Em* – embolus, *Ma* – median apophysis, *Ra* – retrolateral tibial apophysis, *Va* – ventral apophysis. Scale bars: 0.1 mm.

**Female.** Habitus as in Fig. 18E, I, J. Total length 4.95. Carapace 1.95 long, 0.96 wide at pars cephalica, 1.40 wide at pars thoracica. Eye sizes and interdistance of PMEs: AME: 0.15, ALE: 0.12, PME: 0.08, PLE: 0.11, PME–PME: 0.15. Coloration as in male, with paler abdomen lacking a scutum. Measurements of legs: I: 6.54 (1.50, 0.70, 1.44, 1.76, 1.14), II: 6.27 (1.59, 0.67, 1.33, 1.67, 1.01), III: 6.23 (1.60, 0.71, 1.21, 1.74, 0.97), IV: 8.11 (2.04, 0.78, 1.81, 2.46, 1.02).

Epigyne as in Fig. 30A–E. Epigynal plater over 3 times wider than long, lacking fovea; anterior hood wider than long; receptacles with rounded posterior parts, separated by about 4 diameters.

##### Distribution.

Known from the listed localities in Tehran Province, northern Iran (Fig. 33).

#### Acanthinozodium kiana sp. nov.

Taxon classificationAnimaliaAraneaeZodariidae

F7A70D2B-B035-5179-BD17-C0C957A48B7D

http://zoobank.org/820D70DA-633E-4CF3-B11E-3B16C6974CBC

**Figure 24.**
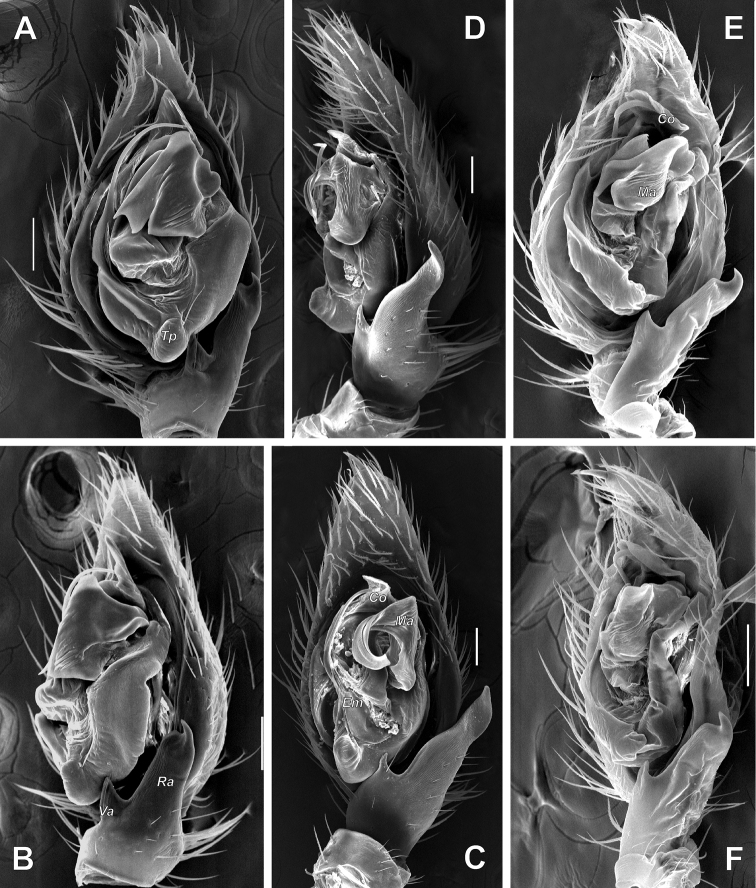
SEM images of the male palps of *Acanthinozodium masa* sp. nov. (**A, B**), *A. dorsa* sp. nov. (**C, D**) and *A. kiana* sp. nov. (**E, F**) **A, C, E** ventral **B, F** retroventral **D** retrolateral. Abbreviations: *Co* – conductor, *Em* – embolus, *Ma* – median apophysis, *Ra* – retrolateral tibial apophysis, *Va* – ventral apophysis, *Tp* – tegular process. Scale bars: 0.1 mm.

Figs 18K , 20G, H , 24E, F , 33

##### Type material.

***Holotype*** ♂ (MHNG), Iran: *Kurdistan Province*: south of Divandareh, 35°45'N, 47°05'E, 23.06.1975 (A. Senglet).

##### Etymology.

The specific epithet is a Kurdish feminine name meaning “nature”. Noun in apposition.

##### Diagnosis.

The new species is very similar to *A. masa* sp. nov. by the shape of the RTA and ventral tibial apophysis but differs by the shape of the prolateral outgrowth of the median apophysis which is finger-like and triangular, terminally with a small hook (*vs.* broad and bifurcated).

##### Description.

**Male.** Habitus as in Fig. 18K. Total length 3.80. Carapace 2.08 long, 1.03 wide at pars cephalica, 1.85 wide at pars thoracica. Eye sizes and interdistance of PMEs: AME: 0.17, ALE: 0.12, PME: 0.09, PLE: 0.10, PME–PME: 0.23. Carapace light brown, darker at pars cephalica. Sternum, labium, maxillae and chelicerae yellowish. Legs yellowish, with numerous macrosetae and without annulations. Abdomen grayish dorsally, lighter ventrally. Spinnerets pale, uniform in color. Measurements of legs: I: 7.57 (2.02, 0.75, 1.67, 1.97, 1.16), II: 7.06 (1.84, 0.74, 1.50, 1.95, 1.03), III: 7.24 (1.98, 0.71, 1.47, 2.08, 1.00), IV: 9.46 (2.63, 0.78, 2.26, 2.77, 1.02).

Palp as in Figs 20G, H, 24E, F. RTA long, with a small projection apically; ventral apophysis relatively small and conical; tegulum with posterior process; median apophysis (*Ma*) wider than long, with a distinct prolateral outgrowth which is triangular terminally and with a small hook; embolus broad basally, originating at about the 7 o’clock position; embolus proper relatively broad and distinctly curved over the median apophysis.

**Female.** Unknown.

##### Distribution.

Known from the type locality in Kurdistan Province, western Iran (Fig. 33).

#### Acanthinozodium masa sp. nov.

Taxon classificationAnimaliaAraneaeZodariidae

675497C0-6927-54C1-8A48-1685DC73BB2B

http://zoobank.org/C50A0614-8086-4BCE-B6D8-8B9E40318781

Figs 18L , 20I–K , 24A, B , 33

##### Type material.

***Holotype*** ♂ and ***paratype*** ♂ (MHNG), Iran: *Kermanshah Province*: Mahi Dasht, 34°14'N, 46°42'E, 29.06.1974 (A. Senglet).

##### Etymology.

The specific epithet is a Kurdish feminine name, meaning “bright like the moon”. Noun in apposition.

##### Diagnosis.

The new species is very similar to *A. diara* sp. nov. by the shape of the RTA, the ventral tibial apophysis and the curvature of the embolus but differs by the shape of the median apophysis (cf. Fig. 20D and 20J) and the conductor lacking a small retrolateral indentation apically (*vs.* present).

##### Description.

Male (holotype). Habitus as in Fig. 18L. Total length 3.05. Carapace 1.47 long, 0.66 wide at pars cephalica, 1.10 wide at pars thoracica. Eye sizes and interdistance of PMEs: AME: 0.12, ALE: 0.10, PME: 0.07, PLE: 0.08, PME–PME: 0.12. Carapace, sternum, labium, chelicerae and maxillae brown; carapace with irregular patterns. Chelicera with one tooth. Legs yellowish brown. Abdomen dorsally black, grayish ventrally. Spinnerets pale, uniform in color. Measurements of legs: I: 5.52 (1.50, 0.47, 1.23, 1.42, 0.90), II: 4.91 (1.15, 0.50, 1.11, 1.30, 0.85), III: 4.62 (1.10, 0.45, 0.94, 1.43, 0.70), Fe IV: 1.80, rest of the segments missing.

Palp as in Figs 20I–K, 24A, B. RTA long and conical, with a small projection apically; tegulum with posterior process (*Tp*); ventral apophysis relatively large and conical; median apophysis wider than long, with a large bifurcated outgrowth prolaterally; embolus broad basally, originating at about the 7 o’clock position; embolus proper thin and steadily curving.

**Female.** Unknown.

##### Distribution.

Known only from the type locality in Kermanshah Province, western Iran (Fig. 33).

#### Acanthinozodium parmida sp. nov.

Taxon classificationAnimaliaAraneaeZodariidae

B7BAE9A0-8746-5000-859F-12DCE23EE8FB

http://zoobank.org/57AB4566-6EB3-4ACF-A4CD-E69F22ECD172

**Figure 25.**
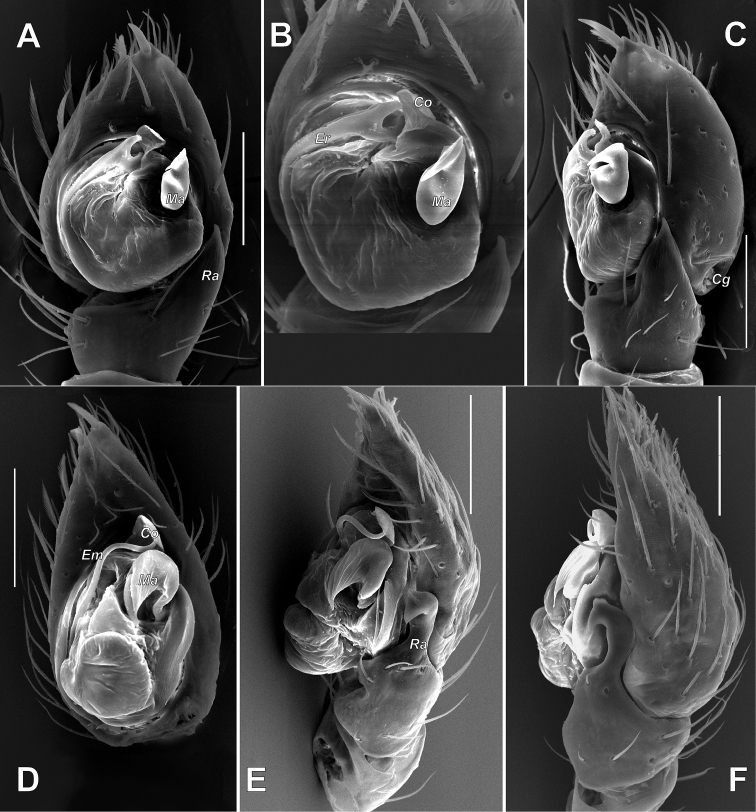
SEM images of the male palps of *Acanthinozodium parmida* sp. nov. (**A–C**) and *Zodarion expers* (**D–F**) **A, B, D** ventral **C, E** retrolateral **F** dorsoretrolateral. Abbreviations: *Cg* – cymbial groove, *Co* – conductor, *Em* – embolus, *Er* – embolar ridge, *Ma* – median apophysis, *Ra* – retrolateral tibial apophysis. Scale bars: 0.1 mm.

Figs 19A–C , 25A–C , 33

##### Type material.

***Holotype*** ♂ (MHNG), Iran: *Isfahan Province*: Qamsar and Barzok Protected Area, 55 km SW of Qamsar, 14 km NE Kamoo, near the road of Gargash observatory, 33°37'N, 51°19'E, 2710 m, 19.05.2016 (P. Ponel).

##### Etymology.

The specific epithet refers to a Persian princess, the only daughter of Bardiya (Smerdis), son of Cyrus the Great. Noun in apposition.

##### Diagnosis.

The new species differs from the congeners in the region by its smaller size, by having a dorsal scutum, and by the very short tip (free part) of embolus having a long furrow with a serrate ventral margin. It is closely related to *A. ovtchinnikovi* sp. nov. from southeastern Turkmenistan, from which it can be differentiated by having an almost as long as wide bulb (*vs.* longer than wide), shorter cymbium (length/width ratio 1.4 vs. 1.86), a different shape of median apophysis (posterior portion larger than anterior one, vs. opposite) and the different position of embolic base (6:30 o'clock, *vs.* 8:00 o'clock; cf. Figs 19B and 22G).

##### Description.

**Male.** Habitus as in Fig. 19A. Total length 1.75. Carapace 0.83 long, 0.42 wide at pars cephalica, 0.60 wide at pars thoracica. Eye sizes and interdistance of PMEs: AME: 0.10, ALE: 0.06, PME: 0.06, PLE: 0.05, PME–PME: 0.10. Carapace, sternum, labium, chelicerae and maxillae yellowish; carapace with irregular dark patterns. Chelicera with one tooth. Legs yellowish, without annulations. Abdomen dorsally black, covered with scutum, ventrally grayish. Spinnerets pale, uniform in color. Measurements of legs: I: 1.89 (0.42, 0.23, 0.41, 0.41, 0.42), II: 1.64 (0.40, 0.23, 0.31, 0.33, 0.37), III: 1.54 (0.41, 0.21, 0.32, 0.29, 0.31), IV: 2.35 (0.60, 0.26, 0.52, 0.59, 0.38).

Palp as in Figs 19B, C, 25A–C. Ventral tibial apophysis lacking, RTA (*Ra*) almost triangular, slightly longer than tibia; cymbium very broad; tegulum slightly wider than long; sperm duct tracking margin of tegulum, lacking any turns; median apophysis (*Ma*) relatively small, less than half of tegulum's height, posterior part larger than anterior; embolus very short, shorter than median apophysis and conductor (*Co*), embolus originating at about 6:30 o'clock position with its terminal 2/3 having a longitudinal furrow (*Er*) with a finely serrated ventral margin.

**Female.** Unknown.

##### Distribution.

Known from the type locality in Isfahan Province, central Iran (Fig. 33).

#### Genus Parazodarion

Taxon classificationAnimaliaAraneaeZodariidae

Ovtchinnikov, Ahmad & Gurko, 2009

F64B37BC-5692-52D1-AA17-139AE84C73BD

##### Type species.

*Zodarion raddei* Simon, 1889 from Turkmenistan.

##### Comments.

Monotypic genus, differing from all other genera in the region by having an elongate cymbium, an S-shaped sperm duct and embolus, a long spine-like outgrowth of the embolic base and an epigyne with large transverse oval fovea.

#### Parazodarion raddei

Taxon classificationAnimaliaAraneaeZodariidae

(Simon, 1889)

6BA12FA2-395B-5701-9392-7134C049CA76

**Figure 26.**
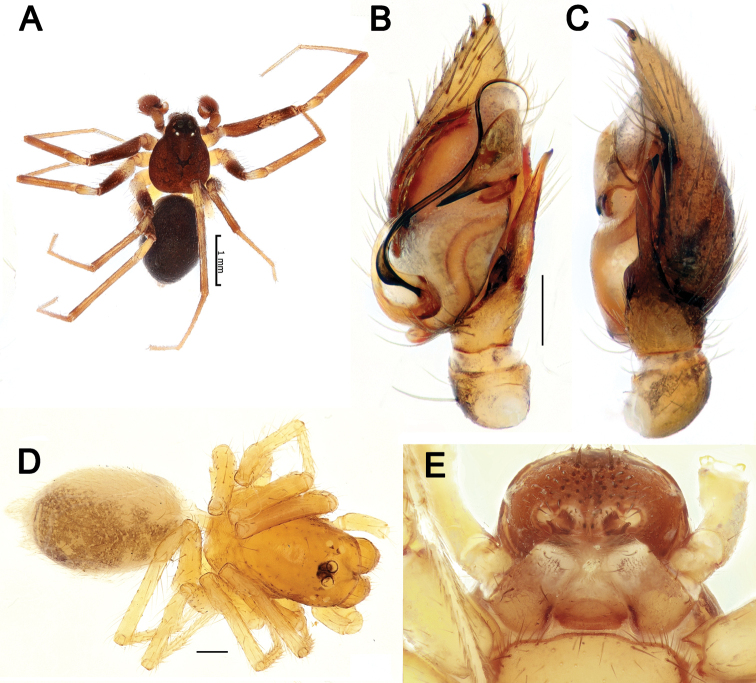
Males of *Parazodarion raddei* (**A–C**) and *Acanthinozodium elburzicum* sp. nov. (**E**) and female of *Trygetus susianus* sp. nov. (**D**) **A, D** habitus, dorsal **B, C** palp, ventral and retrolateral **E** chelicerae, ventral. Scale bars: 0.2 mm, unless stated otherwise.

**Figure 27.**
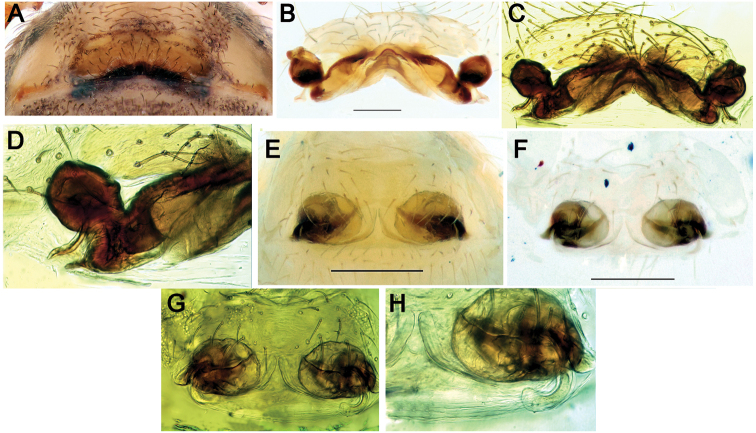
Epigynes of *Parazodarion raddei* (**A–D**) and *Trygetus susianus* sp. nov. (**E–H**) **A, E** intact, ventral **B, F–H** macerated, dorsal **C, D** macerated, ventral. Scale bars: 0.2 mm.

Figs 26A–C , 27A–D , 32

Parazodarion raddei : Ovtchinnikov et al. 2009: 471, f. 1.1–6 (♂♀).
For the complete list of references see WSC (2021).

##### Material.

Iran: *Hamedan Province*: 1♂3♀ (MHNG), around Hamedan, 34°44'N, 48°47'E, 2600 m, 16.06.1975 (A. Senglet); 1♂2♀ (MHNG), Aliabad, 34°51'N, 48°12'E, 02.07.1974 (A. Senglet); *Tehran Province*: 7♂5♀ (ZMMU), 80 km E of Tehran, Damavand area, Aroo, 35°40'N, 52°27'E, 15.06.2000 (Y.M. Marusik & F. Mozaffarian).

##### Records in Iran.

Alborz, Isfahan, Qom, Razavi Khorasan, Sistan and Baluchistan, West Azerbaijan, Yazd, Zanjan (Ovtchinnikov et al. 2009; Hosseini et al. 2014; Sadeghi et al. 2016; Zamani et al. 2017; Zamani et al. 2018; Zamani and Marusik 2018). New records for Hamedan and Tehran (Fig. 32).

##### Records in Turkmenistan.

Ahal, Archman (=Arçman), Ashgabat, Balkan, Lebap, Mary (Simon 1889; Vlassov and Sytshevskaja 1937; Ovtsharenko and Fet 1980; Fet 1985; Mikhailov and Fet 1994; Mikhailov 1997; Ovtchinnikov et al. 2009) (Fig. 32).

##### Distribution.

From United Arab Emirates to Kazakhstan and Afghanistan in the east.

#### Genus Trygetus

Taxon classificationAnimaliaAraneaeZodariidae

Simon, 1882

44378935-A456-55B4-B53C-83F1B267BCA3

##### Type species.

*Palaestina sexoculata* O. Pickard-Cambridge, 1872.

##### Comments.

Small genus with seven named species distributed from Morocco to Turkmenistan (WSC 2021). *Trygetus* differs from all other Zodariinae from the region by having only six eyes (*vs.* eight eyes).

#### Trygetus gromovi

Taxon classificationAnimaliaAraneaeZodariidae

Marusik, 2011

0C488F61-89B4-58B2-8A39-7260E487A64B

Fig. 32

Trygetus gromovi Marusik, 2011: 30, f. 1–7 (♀).

##### Records in Turkmenistan.

Mary (Marusik 2011) (Fig. 32).

##### Distribution.

Known only from the type locality in Turkmenistan.

#### Trygetus susianus sp. nov.

Taxon classificationAnimaliaAraneaeZodariidae

363BA290-2296-556F-82E4-535F556248F1

http://zoobank.org/4E56657F-4A5F-48F2-A3B4-E306128A62E6

Figs 26D , 27E–H , 32

Trygetus jacksoni : Zamani et al. 2018: 188 (♀, misidentified).

##### Type material.

***Holotype*** ♀ (MHNG), Iran: *Khuzestan Province*: north of Andimeshk, 32°41'N, 48°15'E, 17.05.1974 (A. Senglet).

##### Etymology.

This species is named after Susa, one of the most important cities of the Ancient Near East. It is located in the lower Zagros Mountains, about 250 km east of the Tigris River, between the Karkheh and Dez Rivers, in what is currently the Khuzestan Province of Iran.

##### Diagnosis.

The epigyne of the new species is very similar to that of *T. gromovi*. They differ by the presence of a lateral extension of the receptacle and the lack of sclerotized lateral margins in the new species (*vs.* absent and present, respectively).

##### Description.

**Female** (specimen partially bleached). Habitus as in Fig. 26D. Total length 2.07. Carapace 0.96 long, 0.53 wide at pars cephalica, 0.69 wide at pars thoracica. Eye sizes: AME: 0.08, ALE: 0.05, PLE: 0.04. Carapace, sternum, labium, chelicerae and maxillae yellowish brown, without any pattern. Legs slightly lighter than carapace, without spines and without annulations. Abdomen grayish, darker dorsally. Spinnerets pale, uniform in color. Measurements of legs: I: 2.19 (0.61, 0.29, 0.44, 0.44, 0.41), II: 1.98 (0.58, 0.28, 0.39, 0.36, 0.37), III: 1.90 (0.52, 0.26, 0.32, 0.40, 0.40), IV: 2.50 (0.67, 0.28, 0.48, 0.63, 0.44).

Epigyne as in Fig. 27E–H. Epigyne medially with posteriorly diverging sclerotization, laterally with translucent fertilization ducts; receptacles almost round, separated by less than their diameters, each subdivided into a large, weakly sclerotized part and a smaller, more heavily sclerotized part.

**Male.** Unknown.

##### Distribution.

Known from two localities in Khuzestan Province, southwestern Iran (Fig. 32).

#### Genus Zodariellum

Taxon classificationAnimaliaAraneaeZodariidae

Andreeva & Tyshchenko, 1968

A4551295-2A34-50C5-8AD2-BFB995F9BB5C

##### Type species.

*Zodariellum surprisum* Andreeva & Tyshchenko, 1968 from Tajikistan.

##### Comments.

The genus was described as monotypic and synonymized with *Acanthinozodium* by Jocqué (1991). Marusik and Koponen (2001) resurrected *Zodariellum*, described two new species from Mongolia (*Z. schmidti* Marusik & Koponen, 2001 and *Z. mongolicum* Marusik & Koponen, 2001), and transferred eight species to it from *Zodarion*: *Z. asiaticum* (Tyshchenko, 1970), *Z. bekuzini* (Nenilin, 1985), *Z. chaoyangense* (Zhu & Zhu, 1983), *Z. continentalis* (Andreeva & Tyshchenko, 1968), *Z. furcum* (Zhu, 1988), *Z. proszynskii* (Nenilin & Fet, 1985), *Z. nenilini* (Eskov, 1996) and *Z. sytchevskajae* (Nenilin & Fet, 1985). Ponomarev (2007) described two more species in the genus (*Z. volgouralensis* Ponomarev, 2007 and Z. *inderensis* Ponomarev, 2007). Jocqué and Henrard (2015) transferred all *Zodariellum* species, except for the generotype into *Zodarion*.

##### Diagnosis.

All *Zodariellum* spp. are clearly different from the generotype of *Zodarion*, *Z. nitidum* (Audouin, 1826), and the species considered in the genus by the following: 1) the cymbium has a tutaculum (*vs.* lacking in *Z. nitidum* and other species groups); 2) filamentous embolus starting at the 5 o’clock position (*vs.* 6 – in *Z. nitidum*, and many species groups of *Zodarion* sensu lato lack the filamentous embolus, or they are not at 5 o’clock position); 3) the absence of a ventral tibial apophysis (*vs.* present in the generotype) and 4) the shape of the RTA: one arm longer than wide, with claw or wart-like outgrowth posteriorly from the tip (*vs.* wider than long, with 3 branches in the generotype and various shapes in other species groups). Females of the two generotypes have long and twisted copulatory ducts, but they are converging in *Zodariellum* and diverging in *Zodarion*.

##### Composition.

We consider the following species in *Zodariellum* because they have similar male palps and epigynes: *Z. asiaticum* (Tyshchenko, 1970) comb. res., *Z. bactrianum* (Kroneberg, 1875) comb. nov. (ex. *Zodarion*), *Z. bekuzini* (Nenilin, 1985) comb. res., *Z. chaoyangense* (Zhu & Zhu, 1983) comb. res., *Z. continentalis* (Andreeva & Tyshchenko, 1968) comb. res., *Z. furcum* (Zhu, 1988) comb. res., *Z. mongolicum* Marusik & Koponen, 2001 comb. res., *Z. proszynskii* (Nenilin & Fet, 1985) comb. res., *Z. nenilini* (Eskov, 1996) comb. res., *Z. surprisum* Andreeva & Tyshchenko, 1968 comb. res., *Z. schmidti* Marusik & Koponen, 2001 comb. res., *Z. sytchevskajae* (Nenilin & Fet, 1985) comb. res. and *Z. volgouralensis* Ponomarev, 2007 comb. res. Taking into account the shape of the epigyne of *Zodarion inderensis* (Ponomarev, 2007), originally placed in *Zodariellum*, we do not restore the original combination for this species.

To illustrate the conformation of the male palp in *Zodariellum* (male specimens are lacking among the material studied in Iran and Turkmenistan), we have provided figures of *Z. bactrianum*, a species previously known from the original description only and previously considered in *Zodarion*.

##### Distribution.

Western Russia, Iran, Central Asia to northern China (WSC 2021).

#### Zodariellum proszynskii

Taxon classificationAnimaliaAraneaeZodariidae

(Nenilin & Fet, 1985)

B038AB8C-F443-5894-B2AD-946067C7B0E2

Figs 31A–C , 33

Zodarion proszynskii Nenilin & Fet, 1985: 618, f. 1–4, 9 (♂♀).

##### Material.

Iran: *Razavi Khorasan Province*: 1♀ (MHNG), Qouchan, 37°12'N, 58°29'E, 15.07.1974 (A. Senglet).

##### Comparative material.

**Figure 28.**
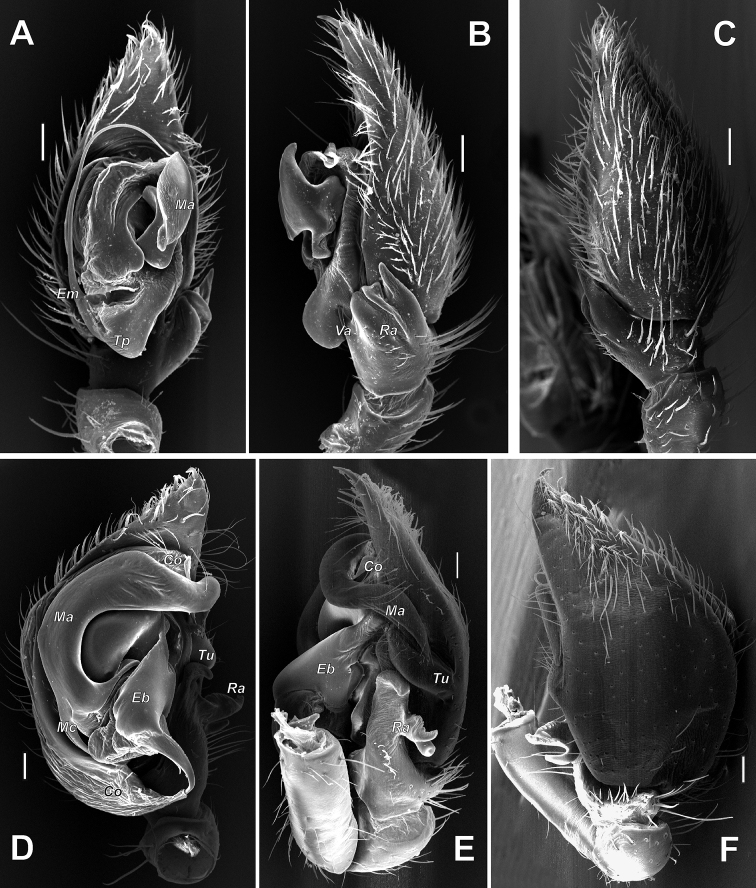
SEM images of the male palps of *Zodarion nitidum* (**A–C**) and *Z. bactrianum* (**D–F**) **A, D** ventral **B, E** retrolateral **C** dorsal **F** retrodorsal. Abbreviations: *Co* – conductor, *Eb* – embolar base, *Em* – embolus, *Ma* – median apophysis, *Mc* – claw of median apophysis, *Ra* – retrolateral tibial apophysis, *Tp* – tegular process, *Tu* – tutaculum, *Va* – ventral apophysis. Scale bars: 0.1 mm.

*Zodariellum bactrianum* (Kroneberg, 1875): 1♂ from Tajikistan (Fig. 28D–F).

**Figure 29.**
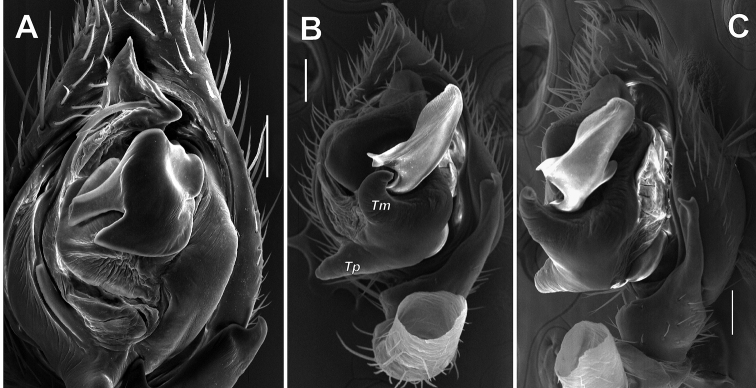
SEM images of the male palps of *Acanthinozodium elburzicum* sp. nov. (**A**) and *Z. talyschicum* (**B, C**) **A, B** ventral **C** ventroretrolateral. Abbreviations: *Tm* – median tegular process, *Tp* – prolateral tegular process. Scale bars: 0.1 mm.

**Figure 30.**
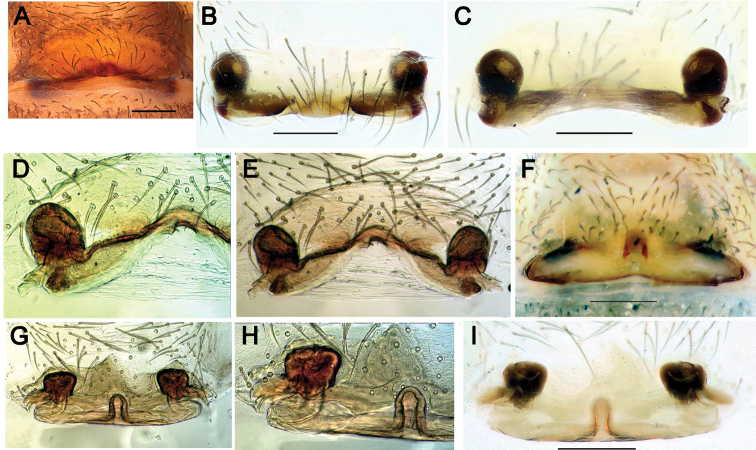
Epigynes of *Acanthinozodium elburzicum* sp. nov. (**A–E**) and *A. dorsa* sp. nov. (**F–I**) **A, F** intact, ventral **B** macerated, ventral **C–E, G–I** macerated, dorsal. Scale bars: 0.2 mm.

**Figure 31.**
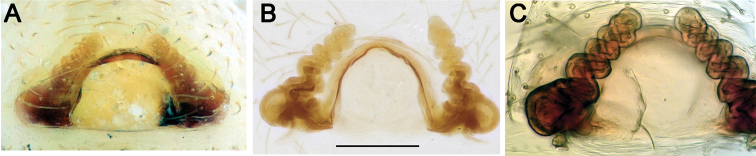
Epigyne of *Zodariellum proszynskii***A** intact, ventral **B, C** macerated, ventral and dorsal. Scale bar: 0.2 mm.

**Figure 32.**
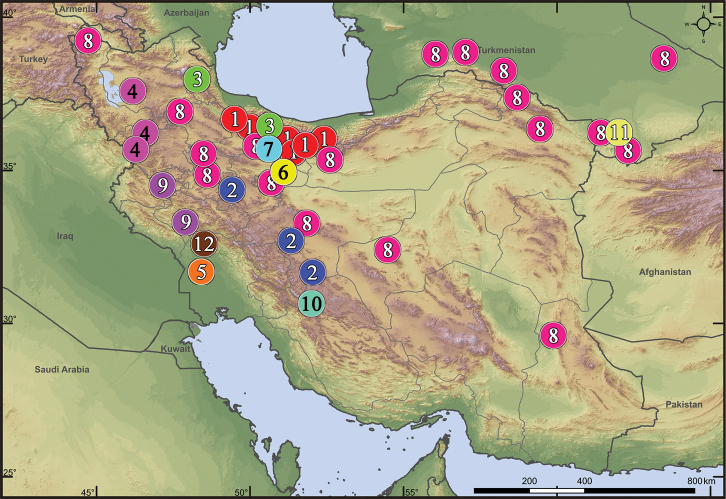
Distribution records of *Acanthinozodium* spp. (partim **1–4, 11**), *Lachesana* spp. (**5–7**), *Parazodarion raddei* (**8**), *Pax* spp. (**9, 10**) and *Trygetus* spp. (**11, 12**) in Iran and Turkmenistan: **1***A. atrisa* sp. nov. **2***A. niusha* sp. nov. **3***A. parysatis* sp. nov. **4***A. sorani* sp. nov. **5***L. insensibilis***6***L. kavirensis* sp. nov. **7***L. perseus* sp. nov. **8***P. raddei***9***P. ellipita* sp. nov. **10***P. leila* sp. nov. **11***A. ovtchinnikovi* sp. nov., *T. gromovi***12***T. susianus* sp. nov.

##### Comments.

This species was previously known from the original description only. It differs from the similar *Z. sytchevskajae* by the shape of the male palpal tibia and the epigyne (see Nenilin and Fet 1985).

##### Records in Iran.

**Figure 33.**
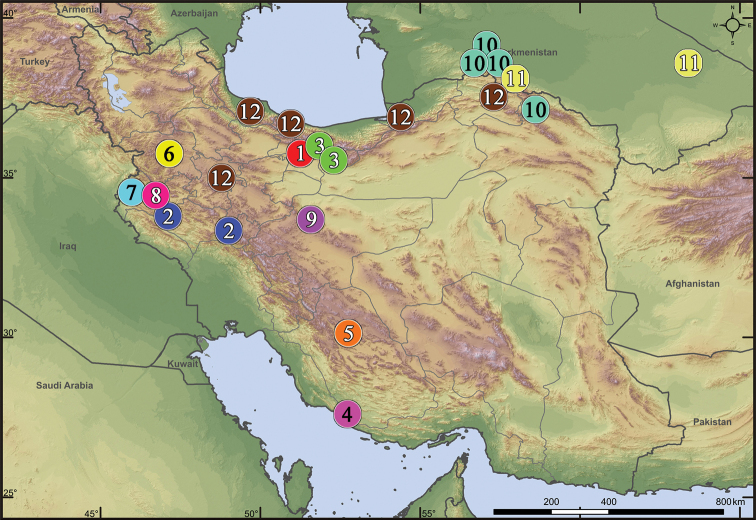
Distribution records of *Acanthinozodium* spp. (partim **1–3, 5, 6, 8, 9**), *Zodariellum* spp. (**10, 11**) and *Zodarion* spp. (**4, 7, 12**) in Iran and Turkmenistan: **1***A. armita* sp. nov. **2***A. diara* sp. nov. **3***A. elburzicum* sp. nov. **4***Z. buettikeri***5***A. dorsa* sp. nov. **6***A. kiana* sp. nov. **7***Z. lutipes***8***A. masa* sp. nov. **9***A. parmida* sp. nov. **10***Z. proszynskii***11***Z. sytchevskajae***12***Z. talyschicum*.

Razavi Khorasan (current data) (Fig. 33).

##### Records in Turkmenistan.

Balkan (Nenilin and Fet 1985) (Fig. 33).

##### Distribution.

Previously known only from the type locality in Turkmenistan. This is a new record for Iran, representing the southernmost record in the known genus range.

#### Zodariellum sytchevskajae

Taxon classificationAnimaliaAraneaeZodariidae

(Nenilin & Fet, 1985)

6A815D86-982B-505E-8552-F43F3F2F2A9E

Fig. 33

Zodarion sytchevskajae Nenilin & Fet, 1985: 619, f. 5–8, 10 (♂♀).

##### Comments.

This species is known from the original description only.

##### Records in Turkmenistan.

Ahal, Lebap (Nenilin and Fet 1985) (Fig. 33).

##### Distribution.

Turkmenistan.

#### Genus Zodarion

Taxon classificationAnimaliaAraneaeZodariidae

Walckenaer, 1826

02249EDF-73C0-59D1-843C-3DE96E998ED5

##### Type species.

*Enyo nitida* Audouin, 1826 from Egypt.

##### Comments.

With 177 named species, this is the largest genus within Zodariinae (WSC 2021). Based on the copulatory organs of the species currently considered in this genus, *Zodarion* does not appear to be monophyletic. There are only two species that are morphologically similar to the generotype: *Z. luctuosum* (O. Pickard-Cambridge, 1872) and *Z. lutipes* (O. Pickard-Cambridge, 1872), from the eastern Mediterranean (east of Tunisia to Iran).

##### Comparative material.

*Zodarion expers* (O. Pickard-Cambridge, 1876), 1♂ from Israel (Figs 19D–F, 25D–F) and *Zodarion nitidum* (Audouin, 1826): 1♂ from Israel (Fig. 28A–C)

##### Comments.

Until recently, *Z. expers* was placed in *Ranops* Jocqué, 1991, but now it is placed in *Zodarion*. It differs from the generotype of *Ranops*, but the copulatory organs are also very different from those of *Z. nitidum*, and most likely it represents a separate genus. Although it is absent in Iran or Turkmenistan, we have provided figures of this species to illustrate the differences with the generotype of *Zodarion*. Furthermore, although *Z. nitidum* has not been recorded in the current study area, because it is the type species of the genus, we have provided illustartions for this species as well, to demonstrate its differences with other Zodariinae genera in Iran and Turkmenistan, as well as with other species currently placed in *Zodarion*.

#### Zodarion buettikeri

Taxon classificationAnimaliaAraneaeZodariidae

(Ono & Jocqué, 1986)

349A2817-76CA-5C7C-B644-621BA3CB3027

Fig. 33

Acanthinozodium buettikeri Ono & Jocqué, 1986: 7, f. 1–4 (♂♀).
Zodarion buettikeri : Levy 1992: 85; Zamani et al. 2017: 69, f. 3E–F (♂).

##### Records in Iran.

Bushehr (Zamani et al. 2017) (Fig. 33).

##### Distribution.

Saudi Arabia, Iran.

##### Comments.

Since the Iranian material of this species has already been illustrated in Zamani et al. (2017), we are not providing new figures for it.

#### Zodarion lutipes

Taxon classificationAnimaliaAraneaeZodariidae

(O. Pickard-Cambridge, 1872)

7906FC6F-1D5A-55C0-8BFE-C19661173E94

Fig. 33

Zodarion lutipes : Bosmans 2009: 281, f. 184–185, 194–195 (♂♀).
For the complete list of references see WSC (2021)

##### Records in Iran.

Kermanshah (Zamani et al. 2018) (Fig. 33).

##### Distribution.

Cyprus, Israel, Lebanon, Jordan, Iran.

##### Comments.

Unfortunately, we were not able to re-examine the material studied by Zamani et al. (2018), therefore, no figures are provided for this species.

#### Zodarion talyschicum

Taxon classificationAnimaliaAraneaeZodariidae

Dunin & Nenilin, 1987

8837EB82-2336-513D-8735-EAA812AFC392

Figs 29B, C , 33

Zodarion talyschicum Dunin & Nenilin, 1987: 196, f. 14–18 (♂♀).
Zodarion talyschicum : Zamani et al. 2020: 589, f. 12D–F (♂♀).

##### Material.

Iran: *Gilan Province*: 1♂ (MHNG), Bidjar, 37°00'N, 49°34'E, 06.09.1973 (A. Senglet); *Golestan Province*: 1♂3♀ (MHNG), Gorgan, Naharkhoran, 36°44'N, 54°29'E, 20.07.1973 (A. Senglet); *Hamedan Province*: 1♂ (NHMW), 10 km SW of Shahpasand, 26.04.1972 (G. Pretzmann); *Mazandaran Province*: 1♂1♀1sub♂ (ZMMU), Barseh Vil., 36°37'N, 50°41'E, 2000 m, 10.06.2000 (Y.M. Marusik); *North Khorasan Province*: 1♂ (MHNG), Bojnurd, 37°29'N, 57°26'E, 26.07.1974 (A. Senglet).

##### Records in Iran.

Golestan (Zamani et al. 2020). New records for Gilan, Hamedan, Mazandaran and North Khorasan, with the latter representing the easternmost record in the whole range (Fig. 33).

##### Distribution.

Azerbaijan, Iran.

## Supplementary Material

XML Treatment for Lachesaninae

XML Treatment for Lachesana

XML Treatment for Lachesana insensibilis

XML Treatment for Lachesana kavirensis

XML Treatment for Lachesana perseus

XML Treatment for Pax

XML Treatment for Pax ellipita

XML Treatment for Pax leila

XML Treatment for Acanthinozodium

XML Treatment for Acanthinozodium atrisa

XML Treatment for Acanthinozodium niusha

XML Treatment for Acanthinozodium ovtchinnikovi

XML Treatment for Acanthinozodium parysatis

XML Treatment for Acanthinozodium sorani

XML Treatment for Acanthinozodium armita

XML Treatment for Acanthinozodium diara

XML Treatment for Acanthinozodium dorsa

XML Treatment for Acanthinozodium elburzicum

XML Treatment for Acanthinozodium kiana

XML Treatment for Acanthinozodium masa

XML Treatment for Acanthinozodium parmida

XML Treatment for Parazodarion

XML Treatment for Parazodarion raddei

XML Treatment for Trygetus

XML Treatment for Trygetus gromovi

XML Treatment for Trygetus susianus

XML Treatment for Zodariellum

XML Treatment for Zodariellum proszynskii

XML Treatment for Zodariellum sytchevskajae

XML Treatment for Zodarion

XML Treatment for Zodarion buettikeri

XML Treatment for Zodarion lutipes

XML Treatment for Zodarion talyschicum
